# Genome-wide identification and evolutionary analysis of the *AP2/EREBP*, *COX* and *LTP* genes in *Zea mays* L. under drought stress

**DOI:** 10.1038/s41598-024-57376-5

**Published:** 2024-03-31

**Authors:** Amaal Maghraby, Mohamed Alzalaty

**Affiliations:** 1 Botany and Microbiology Department, Faculty of Science, Cairo University, Giza, Egypt; 2 Department of Plant Genetic Transformation, Agricultural Genetic Engineering Research Institute (AGERI), Agricultural Research Center (ARC), Giza, Egypt

**Keywords:** *AP2/EREBP*, *COX*, *LTP*, Genome-wide identification, Evolutionary analysis, Drought stress, Biotechnology, Computational biology and bioinformatics, Evolution, Genetics, Molecular biology, Plant sciences

## Abstract

*AP2* (*APETALA2*)/*EREBP* (ethylene-responsive element-binding protein), cytochrome c oxidase (*COX*) and nonspecific lipid transfer proteins (*LTP*) play important roles in the response to drought stress. This is the first study to identify the COX gene in *Zea mays* L*.* via genome-wide analysis. The qRT‒PCR results indicated that *AP2*/*EREBP*, *COX* and *LTP* were downregulated, with fold changes of 0.84, 0.53 and 0.31, respectively, after 12 h of drought stress. Genome-wide analysis identified 78 *AP2/EREBP,* 6* COX* and 10* LTP* genes in *Z. mays* L. Domain analysis confirmed the presence of the AP2 domain, Cyt_c_Oxidase_Vb domain and nsLTP1 in the *AP2/EREBP, COX* and* LTP* proteins, respectively. The *AP2/EREBP* protein family (AP2) includes five different domain types: the AP2/ERF domain, the EREBP-like factor (EREBP), the ethylene responsive factor (ERF), the dehydration responsive element binding protein (DREB) and the SHN SHINE. Synteny analysis of the *AP2/EREBP*, *COX* and *LTP* genes revealed collinearity orthologous relationships in *O. sativa, H. vulgare and A. thaliana*. *AP2/EREBP* genes were found on the 10 chromosomes of *Z. mays* L. *COX* genes were found on chromosomes 1, 3, 4, 5, 7 and 8. *LTP* genes were found on chromosomes 1, 3, 6, 8, 9 and 10. In the present study, the* Ka*/*Ks* ratios of the *AP2/EREBP* paralogous pairs indicated that the *AP2/EREBP* genes were influenced primarily by purifying selection, which indicated that the *AP2/EREBP* genes received strong environmental pressure during evolution. The* Ka*/*Ks* ratios of the *COX-3/COX-4* paralogous pairs indicate that the *COX-3/COX-4* genes were influenced primarily by Darwinian selection (driving change). For the *LTP* genes, the* Ka*/*Ks* ratios of the *LTP-1/LTP-10*, *LTP-5/LTP-3* and *LTP-4/LTP-8* paralogous pairs indicate that these genes were influenced primarily by purifying selection, while the* Ka*/*Ks* ratios of the *LTP-2/LTP-6* paralogous pairs indicate that these genes were influenced primarily by Darwinian selection. The duplication time of the *AP2/EREBP* paralogous gene pairs in *Z. mays* L. ranged from approximately 9.364 to 100.935 Mya. The duplication time of the *COX-3/COX-4* paralogous gene pair was approximately 5.217 Mya. The duplication time of the *LTP* paralogous gene pairs ranged from approximately 19.064 to 96.477 Mya. The major focus of research is to identify the genes that are responsible for drought stress tolerance to improve maize for drought stress tolerance. The results of the present study will improve the understanding of the functions of the *AP2/EREBP*, *COX* and *LTP* genes in response to drought stress.

## Introduction

Drought is defined as dryness (lack of water, moisture deficit, shortage of precipitation) for a period of time that affects organisms (plants, animals, humans) in the affected area^1–4^. Drought is the most destructive type of hydrological hazard^5^. The development of drought-tolerant seed varieties can help farmers produce crops under drought stress. In maize (*Z. mays* L.), drought stress is one of the major environmental stress effects on yield reduction, and drought can affect maize at any stage of development. Breeding programmes are used to improve the drought tolerance of maize hybrids. Molecular biology techniques were used to improve breeding efficiency by identifying genes related to drought stress^6^. Climate changes such as water scarcity have negative effects on plant growth and yield production^7^. Crop plants are exposed to several types of environmental stress, which affects their growth and development throughout their life cycle. Drought activates gene expression pathways in plants to protect cells against water deficit^8^. *APETALA2* (*AP2*)/ethylene-responsive element-binding protein (*EREBP*) is a transcription factor that has essential regulatory functions for protecting plants during stress. AP2/*EREBP* is one of the largest transcription factor families in plants^9–11^. AP2s/EREPs are involved in regulating gene expression during abiotic stress^12^ and plant growth and development^11,13^. The *AP2/EREBP* protein family is classified into: the APETALA2 (*AP2),* ethylene-responsive factor (ERF), dehydration-responsive element binding protein (*DREB*)*,* and related to ABI3/VP1 (*RAV*). In cotton *(Gossypium raimondii*), the genes are distributed on all chromosomes. In *Gossypium hirsutum*, the *ERF* and *DREB* genes play important roles in stress responses^11^. GhERF12 protein play crucial roles in organ development and differentiation in* G. hirsutum*^14^. Cytochrome c oxidase (COX) catalyzes the transfer of electrons from reduced cytochrome c (CYTc) to the final acceptor O_2_ to H_2_O for ATP production^15,16^. Restriction mapping and DNA sequencing were used to study the functional relationships of the *COX* mitochondrial genes. The results did not reveal a transcript of *cox1*; rather, a reduced level of a *cox2* transcript and two different *cox3* transcripts were detected. Results indicate that genomic rearrangements of the both 5' and 3' flanking regions of the *cox1* gene leads to impaired of cox1 transcription^17^. The nonspecific lipid transfer protein (LTPs) are involved in different biological processes and play key roles in plant^18^. *LTP2* play critical role for abiotic^19^ and biotic stresses tolerance in plants^20^. LTPs are a large protein family present in all plants and are expressed in many different tissues. LTPs play important roles in signaling via their structure, which contains a N-terminal signal peptide that delivers proteins to the plasma membrane^21^. In maize, *LTPs* are differentially regulated by drought and salt treatments^22^. In rice, the expression of *LTP* is strongly induced under drought and salinity stresses^23^. In wheat, *TaLTP1.2* and *TaLTP1.13* are upregulated during drought^24^. In *Lotus japonica*, The *LjLTP* genes are expressed in aerial tissues under drought stress^25^. In the Moss Physcomitrium (Physcomitrella) patens, the expression of 8 *LTPgs* was investigated during several abiotic stresses. Three *LTPg* genes are significantly upregulated, which leads to the downregulation of the PpLTPg genes^26^.

## Methods

### Identification of the AP2/EREBP, COX and LTP genes in Z. mays L.

The genomes of *Z. mays* L*., Oryza sativa, Hordeum vulgare* and *Arabidopsis thaliana* were downloaded from the Phytozome database^27^. The *AP2/EREBP* (accession number: NP_001183842.1), *COX* (accession number: NP_001288395.1) and *LTP* (accession number: ABA33850.1) proteins were used as query proteins from the NCBI database (https://www.ncbi.nlm.nih.gov/)^28^ (Sheet 1 Online Resource SI 1) to screen *AP2/EREBP*, *COX* and *LTP* protein members in the genomes of *Z. mays* L*.* from the Phytozome database (https://phytozome.jgi.doe.gov)^27^ with an E-value ≤ 1e^-30^ and ≥ 50% identity for *AP2/EREBP* proteins (https://phytozome-next.jgi.doe.gov/blast-results/694644), whereas the Phytozome database parameters for *COX* proteins (https://phytozome-next.jgi.doe.gov/blast-results/694640) and *LTP* proteins (https://phytozome-next.jgi.doe.gov/blast-results/694645) had an E-value ≤ 1e^−30^.

### Characterization of the AP2/EREBP, COX and LTP proteins in Z. mays L.

Circoletto (http://tools.bat.infspire.org/circoletto/)^29^ visualized the sequence identity of the *AP2/EREBP*, *COX* and *LTP* proteins. The physical and chemical properties of the *AP2/EREBP*, *COX* and *LTP* proteins, including the molecular weight, isoelectric point, total number of negatively charged residues, total number of atoms, instability and grand average hydropathicity (GRAVY), were computed using the ExPASy ProtParam Tool^30^.

### Phylogenetic, chromosomal distribution, evolutionary analysis and synteny analysis of the AP2/EREBP, COX and LTP genes

Multiple sequence alignments of the *AP2/EREBP*, *COX* and *LTP* proteins from *Z. mays* L. were performed via the MUSCLE method. Molecular evolutionary genetic analysis (MEGA-11)^31^ was subsequently conducted on a phylogenetic tree with a maximum likelihood of 1000 bootstrap replicates based on the WAG with Freqs. (+ F) Model. The Itools online website^32^ was used to modify and visualize the tree.

According to the position information of the *AP2/EREBP*, *COX* and *LTP* genes on the chromosome, a karyotype map of the *AP2/EREBP*, *COX* and *LTP* genes was drawn using TBtools^33^. The output image was used to show all the *AP2/EREBP*, *COX* and *LTP* genes on the chromosome.

The rates of synonymous (*Ks*) and nonsynonymous (*Ka*) substitutions were calculated by TBtools^33^ to investigate selection pressure. The divergence time of the gene pairs was estimated using the synonymous mutation rate of substitutions per synonymous site per million years ago (Mya) as follows: “T = Ks/2*λ* × 10^−6^”, with a *λ* value of 6.56 × 10^−9^^34^.

The duplicated genes were identified as paralogous if the alignment covered ≥ 70% of the longer gene and if the identity of the aligned region was ≥ 70%^35^; additionally, the genes were identified by the MEGA-11^31^ gene duplication wizard. Paralogous gene pair (tandem and segmental genes) collinearity analysis was visualized as a Circos plot through TBtools^33^.

TBtools^33^ were used to determine the syntenic relationships of the *AP2/EREBP*, *COX* and *LTP* genes in *Z. mays* L*.* against* O. sativa, H. vulgare* and *A. thaliana*.

### Conserved domain, conserved motif, gene structure and Promoter analyses of the AP2/EREBP, COX and LTP genes

The NCBI conserved domain tool^36^ was used to search against the Pfam v34.0–19,178 PSSMs database for *AP2/EREBP*, *COX* and *LTP* proteins. The InterPro tool^37^ was used to analyze the domains of the *AP2/EREBP*, *COX* and *LTP* proteins. MEME 5.5.2^38^ was used to compute the conserved motifs of the *AP2/EREBP*, *COX* and *LTP* proteins. Pfam^39^ was used for motif description. The gene structures obtained from the GFF file were downloaded from the phytozome of the *Z. mays* L*.* genome and subsequently illustrated using TBtools^33^_._

The promoter sequences of the *AP2/EREBP*, *COX* and *LTP* genes in *Z. mays* L*.*1500 bp upstream of the TSS of each *AP2/EREBP*, *COX* and *LTP* gene were retrieved from the *Z. mays* L*.* genome sequence file and downloaded from the Phytozome database^27^. Cis-regulatory elements (CREs) were also analyzed in Plant CARE^40^. A graphical representation of the CRE elements present in the promoter region of the gene was generated via TBTool^33^.

### Subcellular localization, nuclear localization signal, transmembrane helices, phosphorylation sites and three-dimensional (3-D) structure prediction

Subcellular localization predictor (CELLO) version 2.5 (http://cello.life.nctu.edu.tw/)^41^ was used to predict the subcellular localization of the proteins, and TBtools was used to visualize the results^33^. NLSDB^42^ was used to search for nuclear localization signal potentials. The TMHMM server version 2.0^43^ confirmed the presence of transmembrane helical domains (TMs) in the *AP2/EREBP*, *COX* and *LTP* proteins. The NetPhos 3.1 server^44^ was used to predict the phosphorylation sites of the *AP2/EREBP*, *COX* and *LTP* proteins. The I-TASSER^45^ program predicted the three-dimensional (3-D) structure of the *AP2/EREBP*, *COX* and *LTP* proteins.

### Prediction of miRNAs targeting the *AP2/EREBP*, *COX* and *LTP* genes

The psRNATarget database^46^ and miRBase^47^ were used to predict miRNAs. IPknot^48^ was used to predict RNA secondary structures with pseudoknots for the *AP2/EREBP*, *COX* and *LTP* proteins.

### Gene Ontology enrichment and functional relationship analysis of the AP2/EREBP, COX and LTP genes

ShinyGO 0.77^49^ was used for Gene Ontology enrichment analysis. We performed a gene ontology (GO) annotation analysis by submitting all the *AP2/EREBP*, *COX* and *LTP* gene sequences to the eggNOG database^50^ and Phytozome database^27^. The GO annotation data were processed in SRPLOT^51^ to construct the gene ontology chord for the functional relationships of the *AP2/EREBP*, *COX* and *LTP* genes.

### Maize plant growth and drought treatment

These experiments were conducted in the Department of Botany and Microbiology, Faculty of Science at Cairo University. The seeds used in this study were obtained from the Agricultural Research Center (ARC), located in Giza, Egypt. The seeds originated from Egypt and were certified to be of the white three-way cross 310 variety. Drought treatment was conducted in 2 groups. The first group consisted of control plants, whereas the other consisted of stressed plants. Thirty seeds were planted in small pots in a growth room for 14 days. The second group of stressed plants was subjected to continuous water withholding for 12 h, while the first group was treated with Hoagland’s solution as a control. The plants were harvested after 12 h of drought stress. Three control plants and three stressed plants were subjected to RNA extraction and sequencing.

### RNA isolation, qRT‒PCR expression analysis and sequencing

This study identified *AP2/EREBP* (forward primer: AACCCAAGAACACGCTTCCT and reverse primer: AAGCCACATCCCATCCCAAC), *COX* (forward primer: TCCGTAGTTGGGATTCGTCG and reverse primer: CTGGATTGGTTTCTAGTTTCTTTGA) and *LTP* genes (forward primer: ATAGGAACGTACGCACGCAG and reverse primer: ATGCAAGTCGTGATCATGCG). Total RNA was isolated from the leaves of 15-day-old *Z. mays* L*.* seedlings using a GeneTireX kit. The residual DNA was removed using RNase-free recombinant DNase I (Thermo Scientific, Litwania). First-strand cDNA was synthesized in a 20 μL reaction mixture using a Grisp reverse transcription kit (https://grisp.pt/) with approximately two micrograms of DNA-free total RNA from each sample. qRT‒PCR was performed to quantify the relative transcription levels of the *AP2/EREBP*, *COX* and *LTP* genes expressed in the leaves. qPCR was performed with a CFX Connect Real-Time PCR System (Bio-Rad, Singapore) under the following conditions: 94 °C for 5 min; 40 cycles of 94 °C for 10 s, 58 °C for 20 s, and 72 °C for 30 s; a plate read; a melt curve of 65–95 °C with an increment of 0.5 °C for 10 s; and subsequent sequencing. The Ct (cycle threshold) value was used as a measure of the starting copy number of the target gene^52^. The relative gene expression level was calculated using the 2^−ΔΔ*C*T^ method^53^. Actin (Act) was used as an internal reference gene. The forward primer used was CTGAGGTTCTATTCCAGCCATCC, and the reverse primer used was CCACCACTGAGGACAACATTACC.

### Ethical approval

The authors declare that the experimental research work involving the growth of plants in this study, was conducted in compliance with relevant institutional, national, and international guidelines and legislation.

## Results

### Identification of the AP2/EREBP, COX and LTP genes in Z. mays L.

A total of 78 *AP2/EREBP,* 6* COX* and 10* LTP* candidate genes were retrieved from the *Z. mays* L*.* genome and were named according to their chromosomal positions from *AP2-EREBP*-1 to *AP2-EREBP*-78, *COX-1* to *COX-6* and *LTP*-1 to *LTP*-10 for the *AP2/EREBP*, *COX* and *LTP* genes, respectively (Table S1 Online Resource SI 1).

### Characterization of the AP2/EREBP, COX and LTP proteins in Z. mays L.

**Figure 1:**
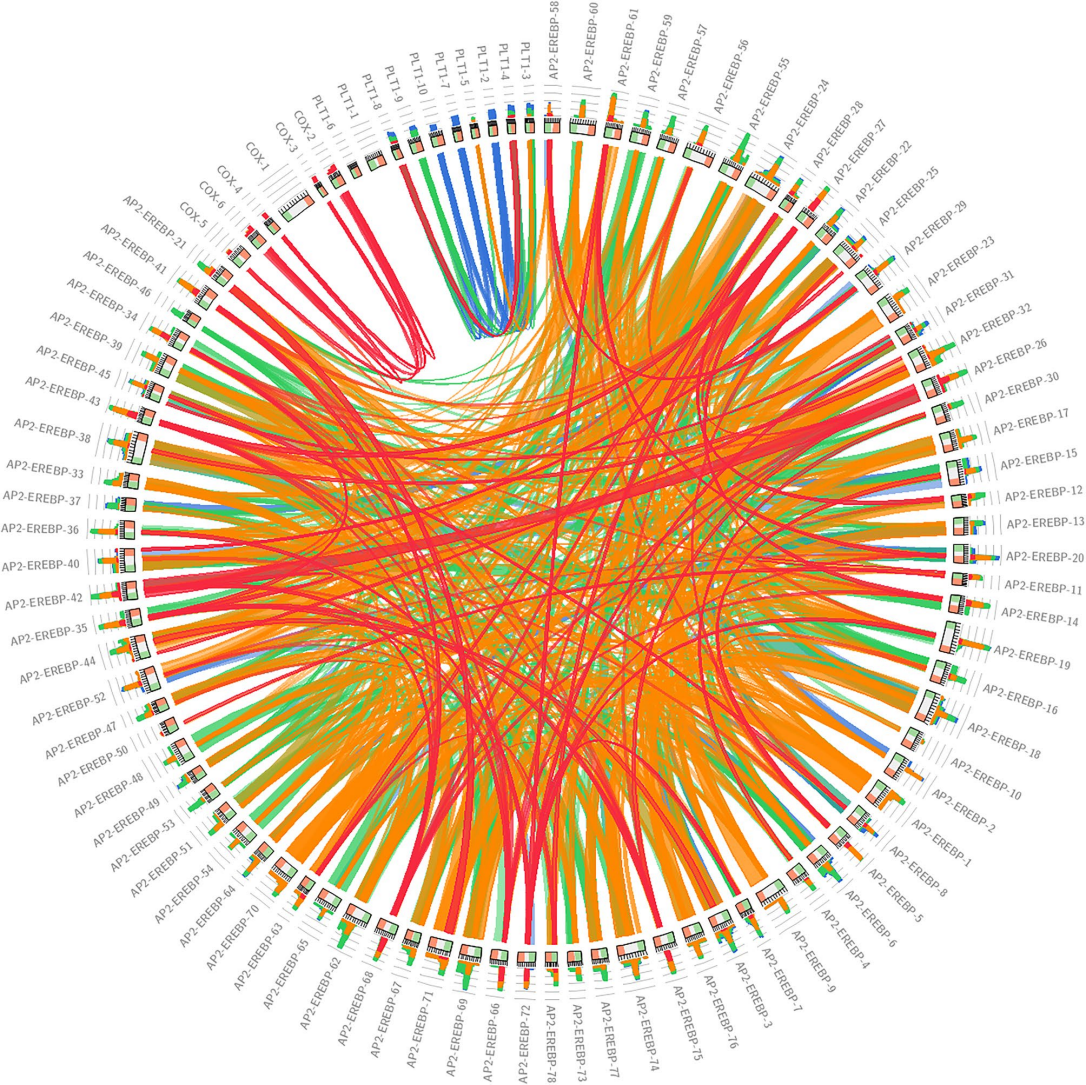
Sequence identity of the *AP2/EREBP, COX* and* LTP* proteins.

The sequence identities of 78 *AP2/EREBP,* 6* COX* and 10* LTP* proteins are shown by the color-by-E-value ratio (blue, ≤ 60%; green, ≤ 80%; orange, ≤ 90%), as shown in Fig. 1. Analysis of protein physical and chemical properties revealed that the length of the *AP2/EREBP* family amino acids in *Z. mays* L*.* ranged from 154 (*AP2-EREBP*-28) to 452 (*AP2-EREBP*-18). The length of the *COX* family amino acids ranged from 88 (*COX*-3) to 483 (*COX*-1). The length of the *LTP* family amino acids ranged from 106 (*LTP*-5) to 247 (*LTP*-1). The molecular weights (MWs) of *AP2/EREBP* ranged from 16837.95 (*AP2-EREBP*-28) to 48127.18 (*AP2-EREBP*-18). The molecular weights of COX ranged from 9972.12 (*COX*-3) to 51,834.35 (*COX*-1). The molecular weights of the* LTPs* ranged from 11062.05 (*LTP*-5) to 24921.63 (*LTP*-1). The isoelectric point (PI) of *AP2/EREBP* ranged from 4.63 (*AP2-EREBP*-30) to 10.31 (*AP2-EREBP*-10). The isoelectric point of COX ranged from 4.46 (*COX*-4) to 10.25 (*COX*-1). The isoelectric point (p-P) of *LTP* ranged from 4.69 (*LTP*-7) to 9.73 (*LTP*-5). The total number of atoms in *AP2/EREBP* ranged from 2329 (*AP2-EREBP*-28) to 6674 (*AP2-EREBP*-24). The total number of atoms in COX ranged from 1361 (*COX*-3) to 7348 (*COX*-1). The total number of atoms in the *LTP* ranged from 1564 (*LTP*-5) to 3509 (*LTP*-1). The average hydropathicity value (GRAVY) of *AP2/EREBP* ranged from − 0.787 (*AP2-EREBP*-13) to − 0.408 (*AP2-EREBP*-58). The average hydropathicity value of COX ranged from − 0.559 (*COX*-6) to 0.231 (*COX*-1). The average hydropathicity value of *LTP* ranged from 0.147 (*LTP*-10) to 0.683 (*LTP*-6) (Table S1 Online Resource SI 1).

### Phylogenetic, chromosomal distribution, evolutionary analysis and synteny analysis of the AP2/EREBP, COX and LTP genes

**Figure 2:**
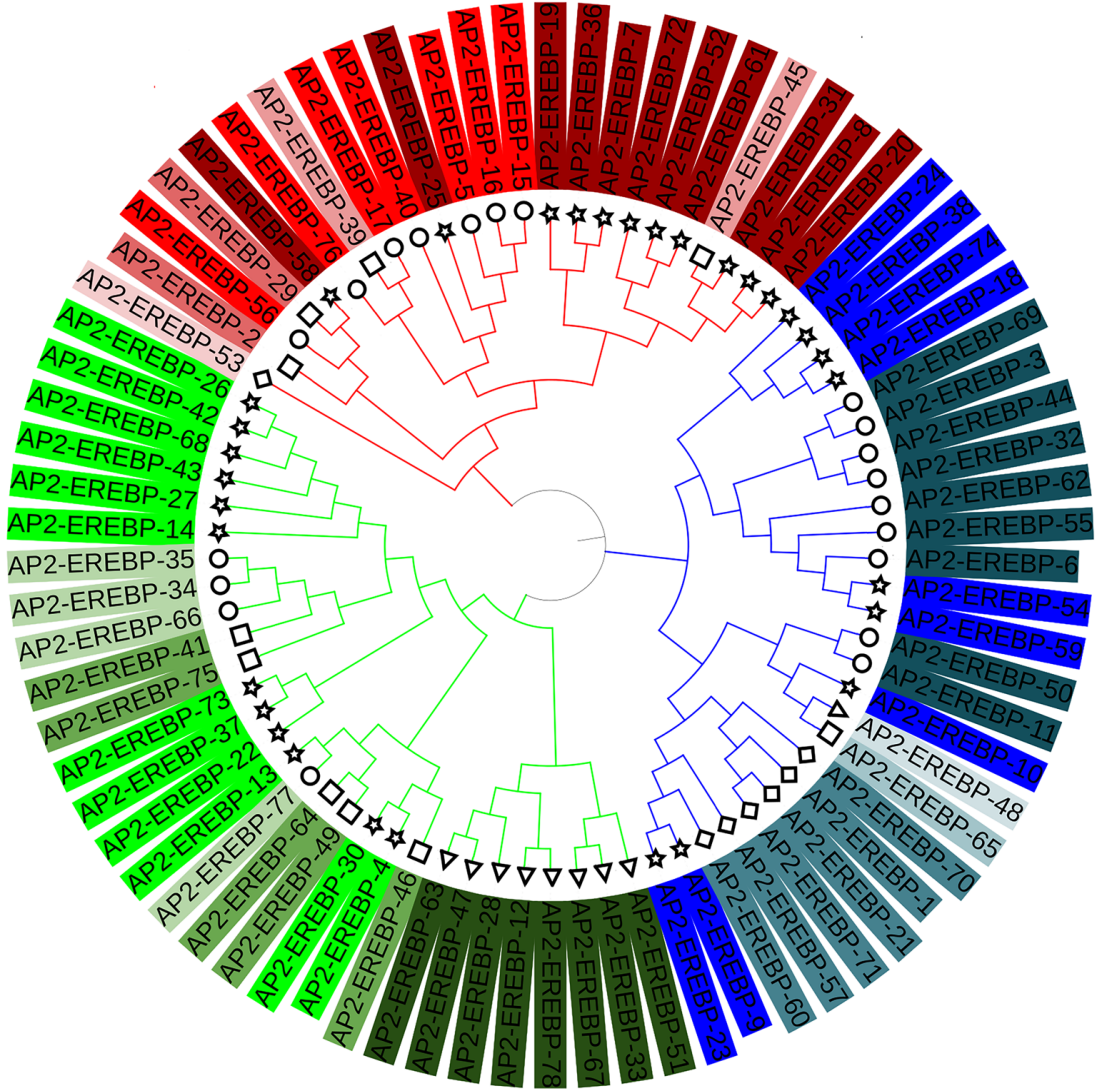
Maximum likelihood phylogenetic tree of the *AP2/EREBP* protein family in *Z. mays* L*.*; the proteins are labeled with ☆ for the AP2/ERF domain, ○ for the EREBP-like factor (EREBP), □ for the ethylene responsive factor (ERF), ◇ for the dehydration responsive element binding protein (DREB) and ◁ for the SHN SHINE.

A phylogenetic tree was constructed using maximum likelihood with 1000 bootstrap replicates, and the *AP2/EREBP*, *COX* and *LTP* protein sequences were used to analyze the possible evolutionary history of *Z. mays* L. In the resulting phylogenetic tree, the *AP2/EREBP* proteins were classified into three distinct clades. The *AP2/EREBP* protein family (AP2) includes five different domain types according to the Phytozome-13 website^27^: the AP2/ERF domain, EREBP-like factor (EREBP), ethylene responsive factor (ERF), dehydration responsive element binding protein (DREB) and SHN SHINE (Fig. 2 and Table S2 Online Resource SI 1). *COX* and *LTP* proteins were classified into three distinct clades (Fig. S1. Online Resource SI 2).

**Figure 3:**
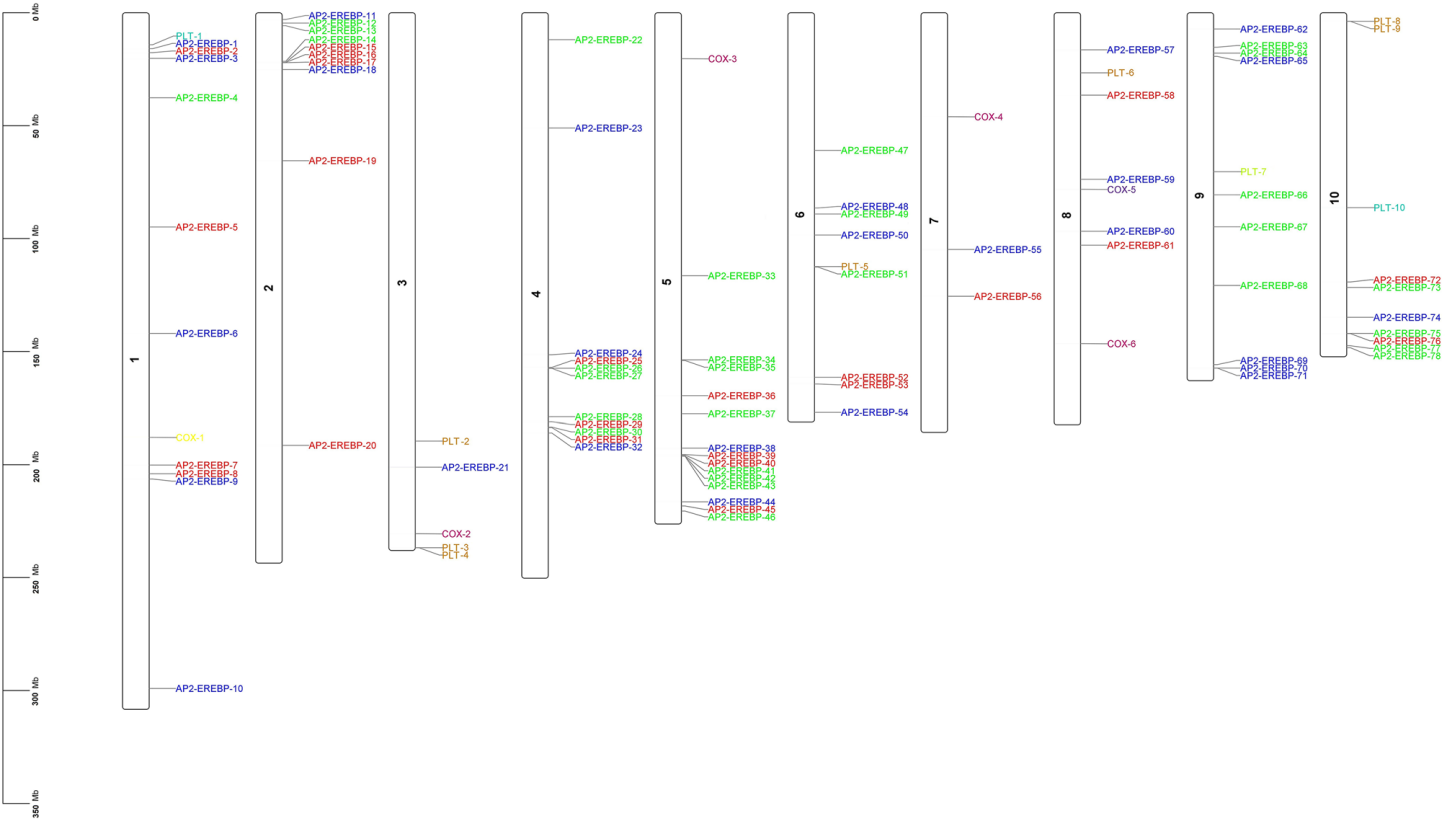
Distribution of the *AP2/EREBP*, *COX* and *LTP* genes on *Z. mays* L*.* chromosomes.

Based on the information available on the Phytozome-13 website^27^, the *AP2/EREBP*, *COX* and *LTP* genes were physically drawn on the chromosomes in the *Z. mays* L*.* genome. *AP2/EREBP* genes were found on 10 chromosomes of *Z. mays* L. *COX* genes were found on chromosomes 1, 3, 4, 5, 7 and 8. *LTP* genes were found on chromosomes 1, 3, 6, 8, 9 and 10 (Fig. 3).

The selective pressure on the *AP2/EREBP*, *COX* and *LTP* genes was investigated by calculating the nonsynonymous/synonymous ratio (*Ka*/*Ks*). A *Ka*/*Ks* ratio > 1 suggested positive selection, a *Ka*/*Ks* ratio = 1 indicated neutral selection, and a *Ka/Ks* ratio < 1 suggested purifying selection^54^. In the present study, the* Ka*/*Ks* ratios of the *AP2/EREBP* paralogous pairs were less than 1, which indicates that the *AP2/EREBP* genes were influenced primarily by purifying selection, which suggests that the *AP2/EREBP* genes received strong environmental pressure during evolution. The* Ka*/*Ks* ratios of the *COX-3/COX-4* paralogous pairs were greater than 1, which indicated that the *COX-3/COX-4* genes were involved in positive or Darwinian selection (driving change). For the *LTP* genes, the* Ka*/*Ks* ratios of the *LTP-1/LTP-10*, *LTP-5/LTP-3* and *LTP-4/LTP-8* paralogous pairs were less than 1, while those of the *LTP-2/LTP-6* paralogous pairs were greater than 1 (Table 1).

**Table 1:** Paralogous pairs of *AP2/EREBP*, *COX* and *LTP* genes and the *Ka*/*Ks* ratio.

Locus 1	locus 2	*Ka*	*Ks*	*Ka*/*Ks*	Time
AP2-EREBP-15	AP2-EREBP-16	0.201887685	0.502399279	0.401847083	38.292628
AP2-EREBP-36	AP2-EREBP-7	0.435204658	0.87977765	0.494675737	67.05622333
AP2-EREBP-52	AP2-EREBP-61	0.07647816	0.207878061	0.367899141	15.84436439
AP2-EREBP-8	AP2-EREBP-20	0.385987339	0.570267544	0.676853071	43.46551404
AP2-EREBP-74	AP2-EREBP-18	0.05168891	0.193976752	0.266469614	14.78481342
AP2-EREBP-69	AP2-EREBP-3	0.077309887	0.187624472	0.412045858	14.30064575
AP2-EREBP-44	AP2-EREBP-32	0.057691935	0.122859443	0.469576728	9.364286818
AP2-EREBP-54	AP2-EREBP-59	0.110298946	0.164779455	0.669373168	12.55940971
AP2-EREBP-50	AP2-EREBP-11	0.338025374	0.624790478	0.541021968	47.62122548
AP2-EREBP-21	AP2-EREBP-71	0.334683458	0.545162686	0.61391483	41.55203397
AP2-EREBP-9	AP2-EREBP-23	0.103291176	0.277490994	0.372232534	21.15022823
AP2-EREBP-33	AP2-EREBP-67	0.176957458	1.324271753	0.133626242	100.935347
AP2-EREBP-47	AP2-EREBP-63	0.128995603	0.294776002	0.437605512	22.46768307
AP2-EREBP-4	AP2-EREBP-30	0.449320876	0.611974544	0.734214978	46.64440122
AP2-EREBP-49	AP2-EREBP-64	0.056604591	0.171835289	0.329411909	13.09720188
AP2-EREBP-37	AP2-EREBP-73	0.290799369	0.632410291	0.459827067	48.2020039
AP2-EREBP-34	AP2-EREBP-35	0.187778474	0.553114724	0.339492814	42.15813446
AP2-EREBP-42	AP2-EREBP-26	0.083190211	0.207698132	0.400534227	15.83065032
COX-3	COX-4	0.100244299	0.068451386	1.464459755	5.217331218
LTP-1	LTP-10	0.80611789	1.265784761	0.636852264	96.477497
LTP-2	LTP-6	0.837292901	0.570546713	1.467527341	43.48679218
LTP-5	LTP-3	0.397424829	0.419284855	0.947863543	31.95768715
LTP-4	LTP-8	0.111949387	0.25012764	0.447569039	19.06460671

**Figure 4:**
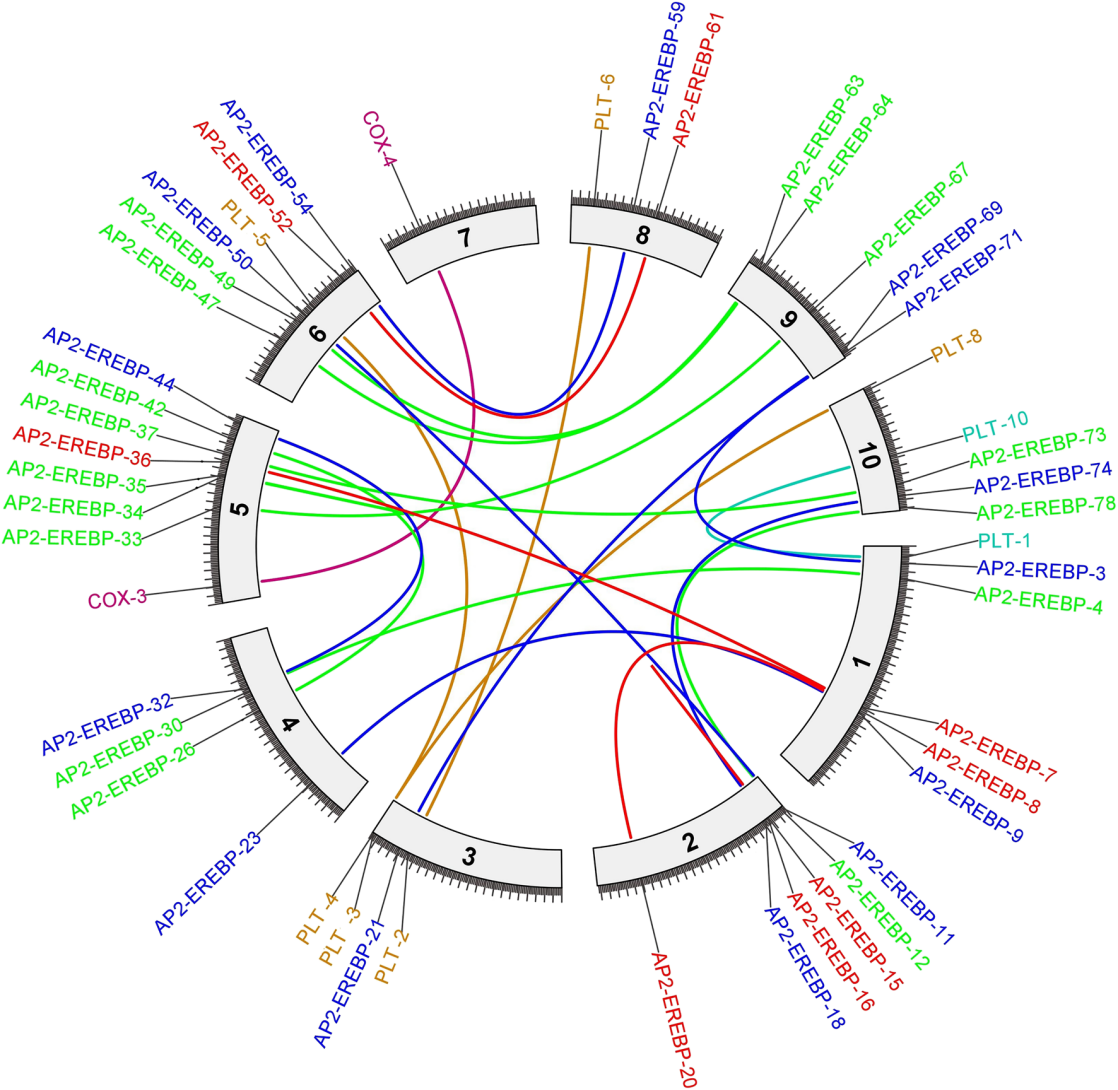
Segmental and tandem duplication of *AP2/EREBP*, *COX* and *LTP* among the *Z. mays* L*.* chromosomes.

The duplication time of the *AP2/EREBP* paralogous gene pairs in *Z. mays* L*.* ranged from approximately 9.364 to 100.935 Mya. The duplication time of the *COX-3/COX-4* paralogous gene pair was approximately 5.217 Mya. The duplication time of the *LTP* paralogous gene pairs ranged from approximately 19.064 to 96.477 Mya (Fig. 4 and Table 1).

**Figure 5:**
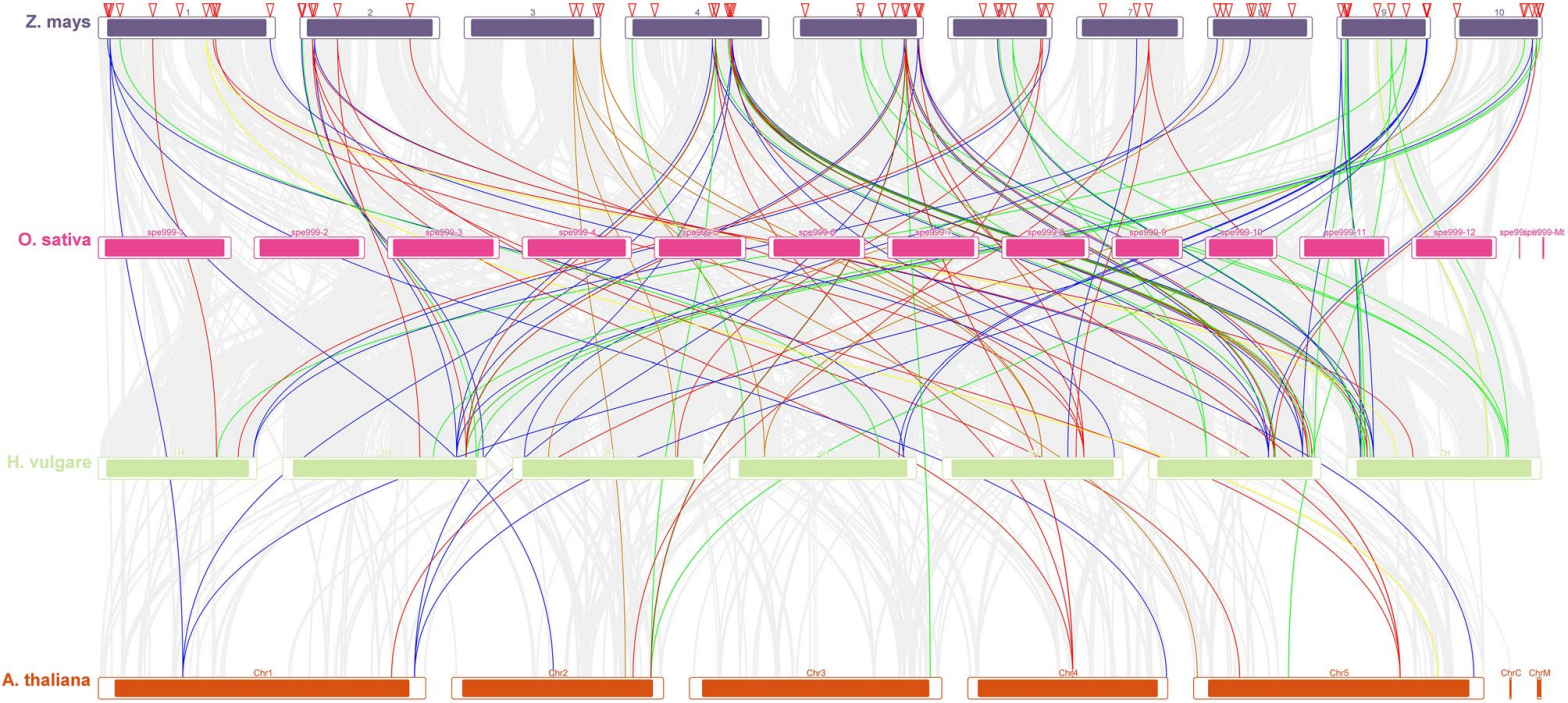
The collinear relationships of the *AP2/EREBP*, *COX* and *LTP* genes are shown as colored lines in the phylogenetic tree.

The *AP2/EREBP*, *COX* and *LTP* genes were analyzed for interspecies collinearity to determine the orthologous relationships of *Z. mays* L*.* with *O. sativa, H. vulgare* and *A. thaliana*. Collinearity analysis revealed robust orthologs of the *AP2/EREBP*, *COX* and *LTP* genes among *Z. mays* L*.* compared with those of the other three plant species (Fig. 5 and Table S3 Online Resource SI 1).

### Conserved domain, conserved motif and gene structure and Promoter analyses of the AP2/EREBP, COX and LTP genes

**Figure 6:**
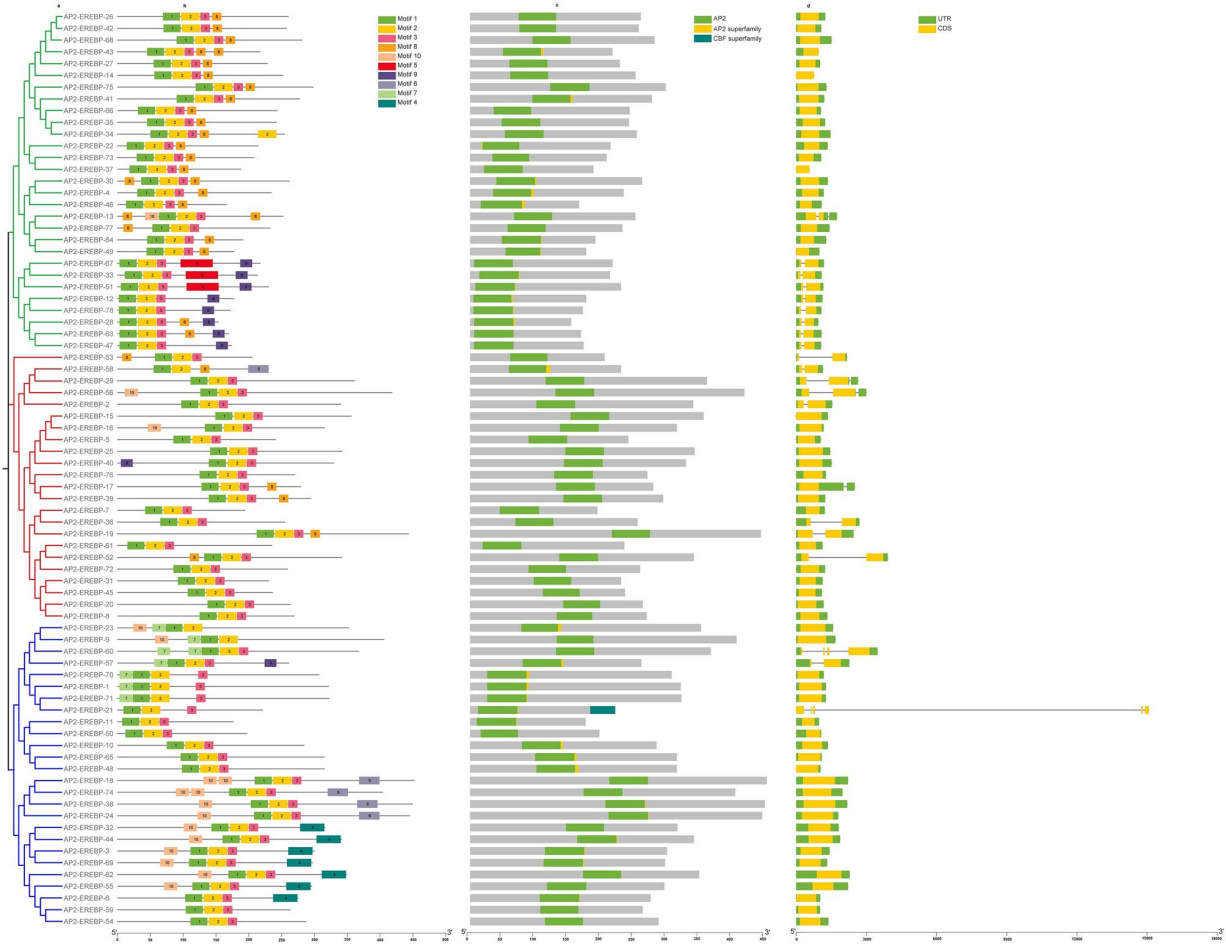
AP2/EREBP proteins. (**a**) Rectangular phylogenetic tree. (**b**) Conserved motifs were predicted using MEME. (**c**) Protein domains. (**d**) Gene structure.

Domain analysis was carried out for all 78 *AP2/EREBP,* 6* COX* and 10* LTP* proteins, and domain analysis confirmed the presence of the AP2 domain (Fig. 6), Cyt_c_Oxidase_Vb domain (Fig. S2 Online Resource SI 2) and the nsLTP1 domain (Fig. S3. Online Resource SI 2) on the *AP2/EREBP, COX* and* LTP* proteins, respectively. Motif analysis indicated that the phylogenetic relationships were similar to the conserved motif distributions within the clade. For instance, the motif distributions of the *AP2/EREBP, COX* and* LTP* proteins exhibited similar motifs within the clade, with few differences. The *AP2/EREBP* motif distributions for *AP2-EREBP*-26, *AP2-EREBP*-42, *AP2-EREBP*-68, *AP2-EREBP*-43, *AP2-EREBP*-27,* AP2-EREBP*-14, *AP2-EREBP*-75, *AP2-EREBP*-41, *AP2-EREBP*-66, *AP2-EREBP*-35, *AP2-EREBP*-34, *AP2-EREBP*-22, *AP2-EREBP*-73, *AP2-EREBP*-37, *AP2-EREBP*-30, *AP2-EREBP*-4, *AP2-EREBP*-46, *AP2-EREBP*-13, *AP2-EREBP*-77, *AP2-EREBP*-64 and *AP2-EREBP*-49 proteins had conserved motif numbers 1, 2, 3, and 8. The *AP2-EREBP*-67, *AP2-EREBP*-33, *AP2-EREBP*-51, *AP2-EREBP*-12, *AP2-EREBP*-78, *AP2-EREBP*-28, *AP2-EREBP*-63 and *AP2-EREBP*-47 proteins carried conserved motif numbers 1, 2, 3, and 9. The remaining *AP2/EREBP* proteins carried conserved motif numbers 1, 2 and 3 (Fig. 6 and Sheet 2 Online Resource SI 1). The *COX* motif distributions for the COX-4, *COX*-3, *COX*-2 and *COX*-6 proteins revealed conserved motif numbers 1 and 3 (Fig. 6 and Sheet 3 Online Resource SI 1). Most of the *LTP* proteins presented conserved motif numbers of 1, 2 and 3 (Fig. 6 and Sheet 4 Online Resource SI 1). The exon‒intron structure is an important source of plant biodiversity and gene family evolution. The gene structure results revealed that 21 of the 78* AP2/EREBP* genes had introns (Fig. 6). All the *COX* genes had introns, while 8* LTP* genes had introns (Fig. S2 and Fig. S3 Online Resource SI 2).

**Figure 7:**
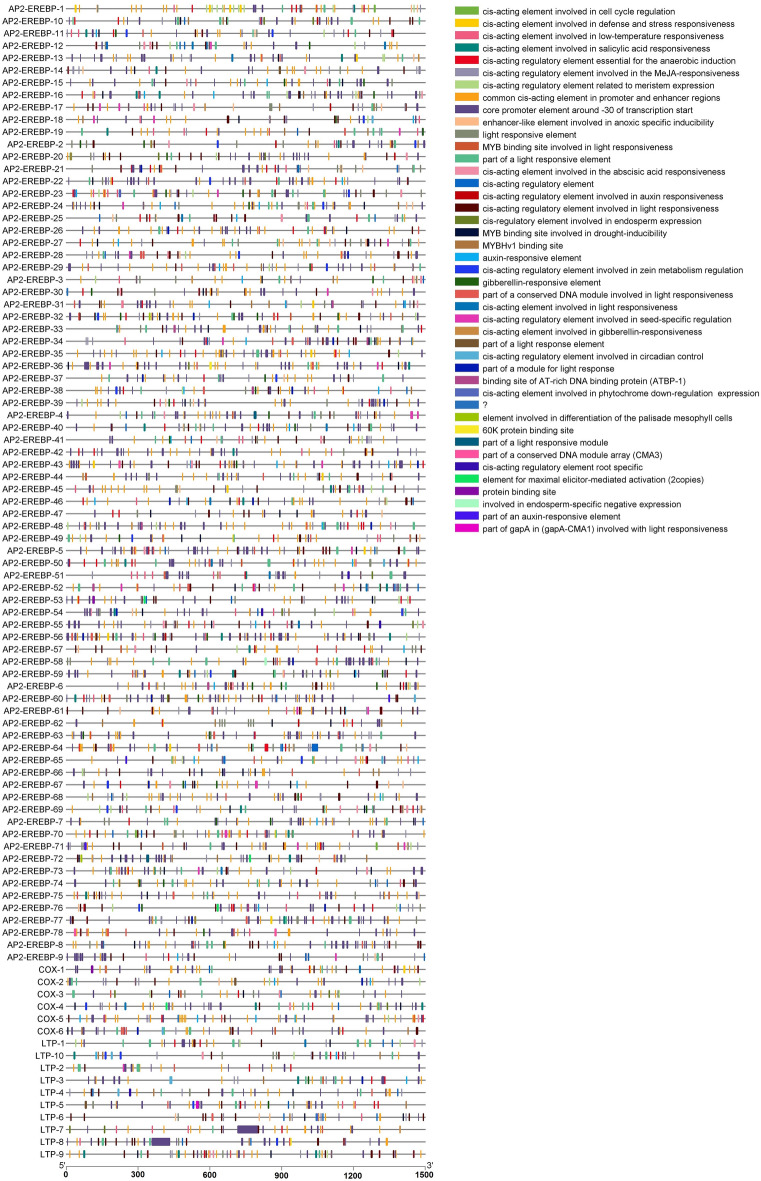
Cis‐acting elements in the promoter regions (1500 bp upstream of the start codon) of the *AP2/EREBP*, *COX* and *LTP* genes*.*

The *AP2/EREBP*, *COX* and *LTP* gene sequences (1500 bp upstream of the start codon) (Table S4 Online Resource SI 1) were selected for cis-element analysis using the PlantCARE web tool to identify their biological functions (stress response, growth and development). The promoter regions of the *AP2/EREBP*, *COX* and *LTP* genes in *Z. mays* L*.* contain a large number of plant hormone response elements. Most *AP2/EREBP*, *COX* and *LTP* proteins contain defense and stress response elements, abscisic acid-responsive elements, methyl jasmonate (MeJA)-responsive elements, salylic acid and the MYB binding site (MBS) element, which are involved in the drought response (Fig. 7).

### Subcellular localization, nuclear localization signal, transmembrane helices, phosphorylation sites and three-dimensional (3-D) structure prediction

Subcellular localization analysis revealed that most *of* the *AP2/EREBP* proteins were located in the nucleus. *COX* proteins were predicted to be expressed in different organelles; for instance, COX-1 was predicted to be expressed in the plasma membrane, whereas COX-2 and COX-6 were predicted to be expressed in chloroplasts. Most of the *LTP* proteins were located in the extracellular space. A heatmap was constructed to predict the subcellular localization of the *AP2/EREBP, COX* and* LTP* proteins, as shown in Fig. S4 Online Resource SI 2 and Table S5 Online Resource SI 1.

Sixteen putative nuclear localization signals (NLSs) were predicted for 21* AP2/EREBP* proteins_,_ whereas no nuclear localization signals (NLSs) were predicted for* COX* or* LTP* proteins (Table S6 Online Resource SI 1).

The TMHMM results predicted the transmembrane helices in AP2-EREBP-10, AP2-EREBP-21, COX-1 and all 10 LTP proteins (Fig. S5, S6 and S7 Online Resource SI 2).

The phosphorylation site prediction results for the AP2/EREBP, COX and LTP proteins for kinases are shown in Table S7 Online Resource SI 1.

**Figure 8:**
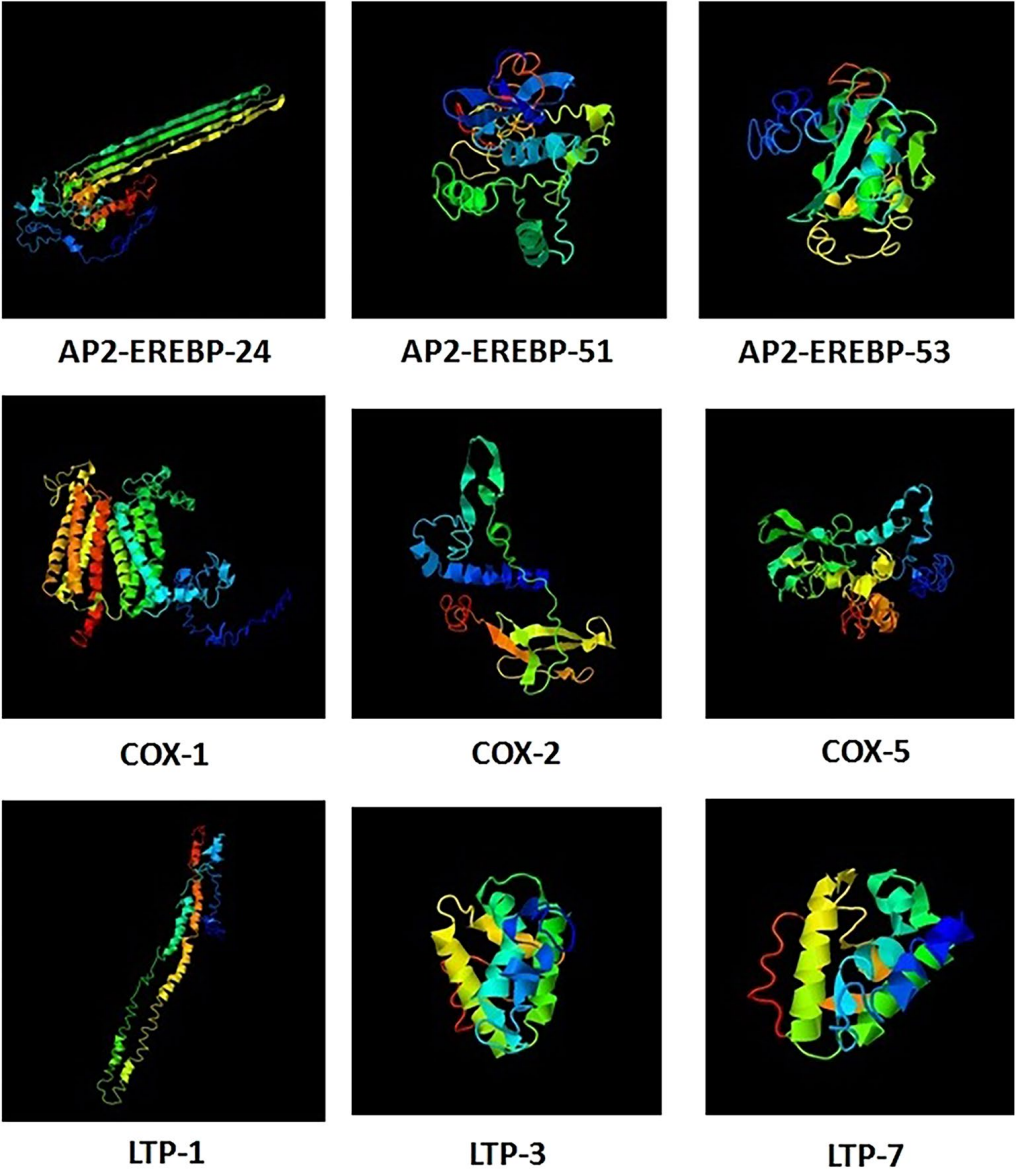
Structural analysis of the AP2-EREBP-24, AP2-EREBP-51, AP2-EREBP-53, COX-1, COX-2, COX-5, LTP-1, LTP-3 and LTP-7 proteins.

To study the putative functions of the AP2/EREBP, COX and LTP proteins in *Z. mays* L*.*, we selected a protein from each clade. The AP2-EREBP-24, AP2-EREBP-51, AP2-EREBP-53, COX-1, COX-2, COX-5, LTP-1, LTP-3 and LTP-7 proteins were modeled with I-TASSER software to construct 3-D structures. The 3-D structures were constructed according to similar structural templates and crystal structures obtained from the Protein Data Bank (Fig. 8). C-scores were used to estimate the confidence of the constructed protein model for the AP2-EREBP-24, AP2-EREBP-51, AP2-EREBP-53, COX-1, COX-2, COX-5, LTP-1, LTP-3 and LTP-7 proteins. The closest structural similarity protein models were selected as the best-predicted models for the AP2/EREBP, COX and LTP proteins, with C-scores ranging (Table 2). Due to their structural similarity, proteins that are structurally close to the target in the PDB often have similar functions. The C-scores suggested that the structures of the AP2/EREBP, COX and LTP proteins were constructed with high accuracy.

**Table 2:** Modeling parameters for the AP2/EREBP, COX and LTP proteins.

Protein	C-Score	TM-Score	RMSD (Å)	Best Identified Structural Analogs in PDB				
				PDB Hit	TM-Score a	RMSD a	IDEN a	Cov
AP2-EREBP-24	− 1.98	0.48 ± 0.15	11.8 ± 4.5	5fmwA	0.896	2.59	0.094	0.957
AP2-EREBP-51	− 3.82	0.30 ± 0.10	14.9 ± 3.6	6fokA	0.438	5.86	0.053	0.748
AP2-EREBP-53	− 2.77	0.40 ± 0.13	11.8 ± 4.5	5wx9A	0.561	2.35	0.315	0.634
COX-1	− 1.50	0.53 ± 0.15	10.8 ± 4.6	6a2jA	0.623	1.12	0.154	0.631
COX-2	− 2.04	0.47 ± 0.15	9.5 ± 4.6	6t15d	0.690	1.18	0.283	0.727
COX-5	− 4.30	0.26 ± 0.08	16.4 ± 3.0	6rc9A1	0.435	6.24	0.063	0.771
LTP-1	− 4.31	0.26 ± 0.08	16.5 ± 3.0	5owvC	0.536	4.62	0.044	0.757
LTP-3	− 1.19	0.57 ± 0.15	6.8 ± 4.1	4xuwA	0.718	0.88	0.446	0.748
LTP-7	− 0.89	0.60 ± 0.14	6.1 ± 3.8	4xuwA	0.720	1.08	0.319	0.765

### Prediction of miRNAs targeting the AP2/EREBP, COX and LTP proteins

A total of 187 microRNAs were predicted to target the AP2/EREBP genes, 48 microRNAs were predicted to target COX proteins, and 30 microRNAs were predicted to target LTP genes. The microRNA targeting relationships for the AP2/EREBP, COX and LTP genes are shown in Table S8 Online Resource SI 1.

The results from the prediction of RNA secondary structures with pseudoknots for the AP2/EREBP (AP2-EREBP-24, AP2-EREBP-51 and AP2-EREBP-53); COX (COX-1, COX-2 and COX-5); and LTP (LTP-1, LTP-3 and LTP-7) proteins are shown in Fig. S8: Fig. S16 Online Resource SI 2.

### Gene ontology enrichment and functional relationship analysis

To further determine the functions of the *AP2/EREBP*, *COX* and *LTP* genes, we performed enrichment analysis and gene ontology (GO) analysis based on biological processes and molecular functions. GO terms help us understand the function of genes at the molecular level (Figs. S17, S18 and S19 Online Resource SI 2). GO terms for the *AP2/EREBP*, *COX* and *LTP* genes confirmed the functional role of *AP2/EREBP*, *COX* and *LTP* as stress responsive genes (Fig. S20, S21 and S22 Online Resource SI 2).

In the present study, qRT‒PCR analysis revealed that the *AP2/EREBP*, *COX* and *LTP* proteins were expressed in leaves, and drought decreased the expression levels of *AP2/EREBP*, *COX* and *LTP* by 0.84, 0.53 and 0.31, respectively, after 12 h of drought stress (Sheet 1 Online Resource SI 1). Domain structure, promoter and gene ontology enrichment analyses confirmed the functional role of the *AP2/EREBP*, *COX* and *LTP* proteins in stress responses.

## Discussion

Genome-wide analysis identified 78 *AP2/EREBP* genes in *Z. mays* L. Phylogenetic classification revealed that the 78 *AP2/EREBP* proteins could be divided into three distinct clades, which included the AP2/ERF domain, the EREBP-like factor (EREBP), the ethylene responsive factor (ERF), the dehydration responsive element binding protein (DREB) and the SHN SHINE according to the description available on Phytozome-13^27^. Cheng *et al.*^55^ identified 229 *AP2/ERF* genes in the maize genome. In addition, phylogenetic analysis revealed that the *ZmAP2*/*ERF* family members could be divided into five clades, namely, 27 *AP2* (*APETALA2*), 105 *ERF* (*ethylene responsive factor*), 89 *DREB* (*dehydration responsive element binding*), 5 *RAV* (*related to ABI3*/*VP*) and a soloist. In this study, we identified 6 *COXs* in *Z. mays* L. To date, no comprehensive investigation of the COX gene in maize has been reported via genome-wide analysis. We also identified 10* LTP* genes in *Z. mays* L*.* Wei and Zhong^22^ identified 63 *LTP* genes in maize, which were divided into six types, whereas Fang *et al.*^56^ identified 65 LTP genes in maize. Our qRT‒PCR results indicated that *AP2/EREBP COX* and *LTP* were downregulated, with fold changes of 0.84, 0.53 and 0.31, respectively, under drought stress. Sharoni *et al.*^57^ reported the same results for the AP2, DREB, and ERF genes in the IR77298-14-1-2-B-10 line; these genes were highly activated in leaves under severe stress treatment and downregulated under severe stress treatment. Similarly, Trindade *et al.*^58^ reported the same results in *Medicago truncatula* for *COX5b*, which strongly downregulated under water deficit conditions. Wei and Zhong^22^ reported the same results in maize, in four* ZmLTP* which were downregulated under drought stress. These results suggest that, under drought stress, *ZmLTP* genes may exhibit tissue-specific expression because *ZmLTP1.2* was significantly downregulated in ovarian tissue but upregulated in the leaf meristem. In the present study, Pfam domain analysis confirmed the presence of the AP2 domain, Cyt_c_Oxidase_Vb and nsLTP1 on the *AP2/EREBP*, *COX* and *LTP* proteins, respectively. Motif and gene structure analyses indicated that genes with closer phylogenetic relationships exhibited more similar genetic structures. The promoter regions of the *AP2/EREBP*, *COX* and *LTP* genes contain defense and stress response elements, abscisic acid-responsive elements, methyl jasmonate (MeJA)-responsive elements, salylic acid and the MYB binding site (MBS) element, which are involved in the drought response. The *AP2/EREBP* genes were found on 10 chromosomes of *Z. mays* L*.*, the same results were found by Cheng *et al*^55^. *COX* genes were found on chromosomes 1, 3, 4, 5, 7 and 8. *LTP* genes were found on chromosomes 1, 3, 6, 8, 9 and 10. The* Ka*/*Ks* ratios of the *AP2/EREBP* paralogous pairs were less than 1, which indicates that the *AP2/EREBP* genes were influenced primarily by purifying selection, which means that the *AP2/EREBP* genes received strong environmental pressure during evolution. The* Ka*/*Ks* ratios of the *COX-3/COX-4* paralogous pairs were greater than 1, which indicated that the *COX-3/COX-4* genes were involved in positive or Darwinian selection (driving change). For the *LTP* genes, the* Ka*/*Ks* ratios of the *LTP-1/LTP-10*, *LTP-5/LTP-3* and *LTP-4/LTP-8* paralogous pairs were less than 1, which indicates that these genes were involved in purifying selection, while the Ka/Ks ratios of the *LTP-2/LTP-6* paralogous pairs were greater than 1. Synteny analysis of the *AP2/EREBP*, COX and LTP proteins revealed collinearity orthologous relationships in *O. sativa, H. vulgare and A. thailana*. Gene Ontology enrichment analysis confirmed the functional role of stress-responsive *AP2/EREBP*, COX and LTP.

## Conclusion

This is the first study to identify the COX gene in *Z. mays* L*.* by genome-wide analysis. Domain structure, promoter and gene ontology enrichment analyses confirmed the functional role of the *AP2/EREBP*, *COX* and *LTP* proteins in stress responses. The results of the present study could improve the understanding of how *AP2/EREBP*, *COX* and *LTP* are mechanistically linked to drought stress responses in maize and could be used for the genetic improvement of maize.

## Supplementary Information

Supplementary Tables.

Supplementary Figures.

## Data Availability

All data generated or analyzed during this study are included in this published article and its supplementary information files.

## Abbreviations

*AP2/EREBP*: *APETALA2*/Ethylene-responsive element-binding protein
*COX*: Cytochrome c oxidase
*LTP*: Nonspecific lipid transfer protein
Ka/*Ks*: Ratio of nonsynonymous/synonymous
RMSD: Root mean square deviation
