# Correction: Genome-wide identification and evolutionary analysis of the *AP2/EREBP*, *COX* and *LTP* genes in *Zea mays* L. under drought stress

**DOI:** 10.1038/s41598-026-41866-9

**Published:** 2026-03-09

**Authors:** Amaal Maghraby, Mohamed Alzalaty

**Affiliations:** 1 Botany and Microbiology Department, Faculty of Science, Cairo University, Giza, Egypt; 2 Department of Plant Genetic Transformation, Agricultural Genetic Engineering Research Institute (AGERI), Agricultural Research Center (ARC), Giza, Egypt

Correction to: *Scientific Reports* 10.1038/s41598-024-57376-5, published online 31 March 2024

The original version of this Article contained an error in the Methods section, under the subheading ‘Phylogenetic, chromosomal distribution, evolutionary analysis and synteny analysis of the AP2/EREBP, COX and LTP genes’.

“The divergence time of the gene pairs was estimated using the synonymous mutation rate of substitutions per synonymous site per million years ago (Mya) as follows: “T = Ks/2*λ*”, with a *λ* value of 6.05 × 10^−9^^34^.”

now reads:

“The divergence time of the gene pairs was estimated using the synonymous mutation rate of substitutions per synonymous site per million years ago (Mya) as follows: “T = Ks/2*λ* × 10^−6^”, with a *λ* value of 6.56 × 10^−9^^34^.”

In addition, Reference 34, “Yang, S., Zhang, X., Yue, J.-X., Tian, D. & Chen, J.-Q. Recent duplications dominate NBS-encoding gene expansion in two woody species. *Mol. Genet. Genom.* 280, 187–198. 10.1007/s00438-008-0355-0 (2008)” was incorrectly cited in the Article and listed in the Reference list. This reference has now been removed and replaced with the correct citation of Reference 34: “He, Y. et al. Genome-Wide Identification and Expression Analysis of Two-Component System Genes in Tomato. *Int. J. Mol. Sci.* **2016**, *17*, 1204. 10.3390/ijms17081204.”

The original Article has been corrected.

