# Supplementary material for: Genome-wide identification and evolutionary analysis of the AP2/EREBP, COX and LTP genes in Zea mays L. under drought stress
Source: Sci Rep. 2024 Mar 31;14:7610. doi: 10.1038/s41598-024-57376-5 (PMC10982304; doi:10.1038/s41598-024-57376-5)
Supplement: Supplementary file 2 — Supplementary Figures. [file 41598_2024_57376_MOESM2_ESM.pdf]

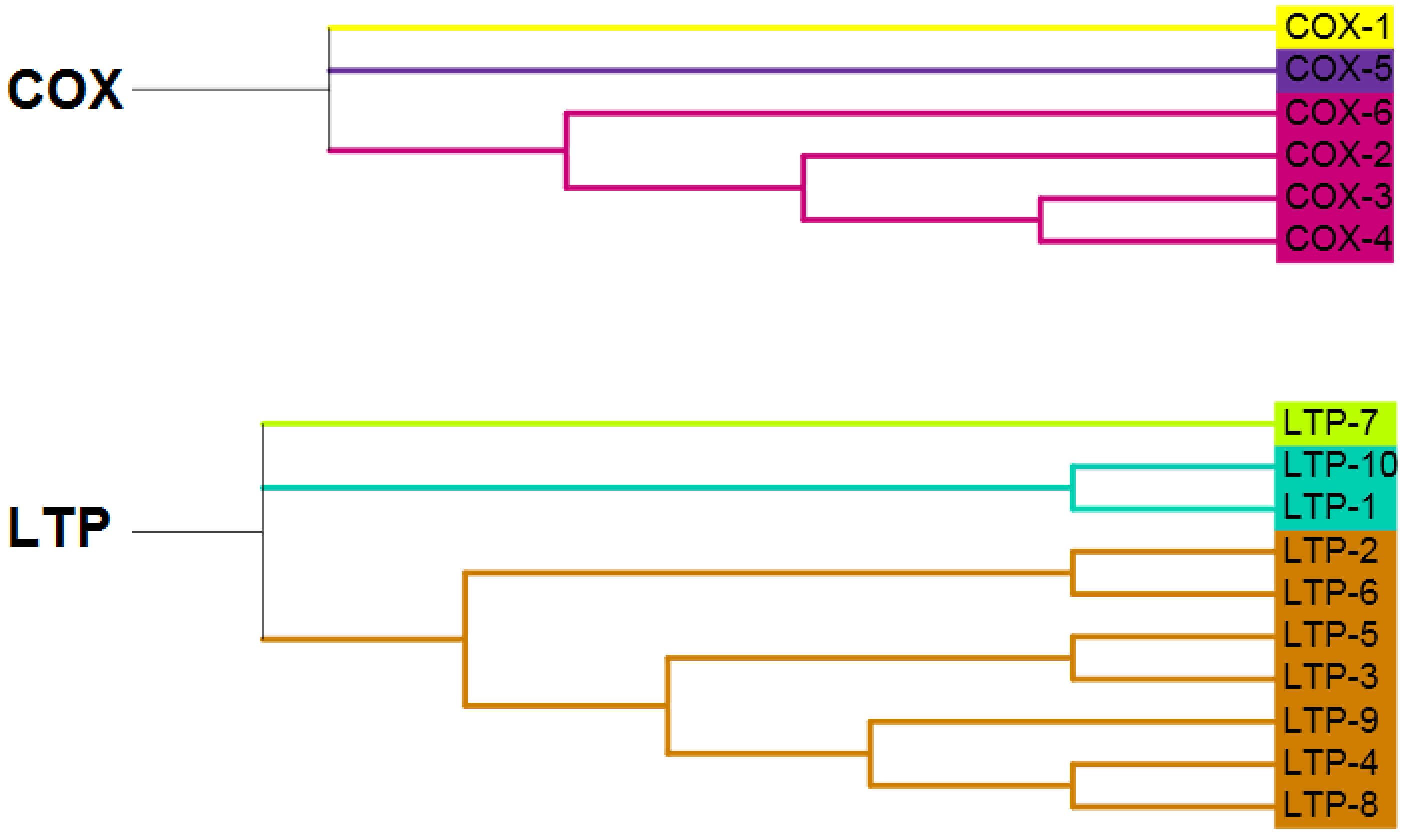

Fig. S1. Maximum likelihood phylogenetic tree of the COX and LTP proteins in Z. mays

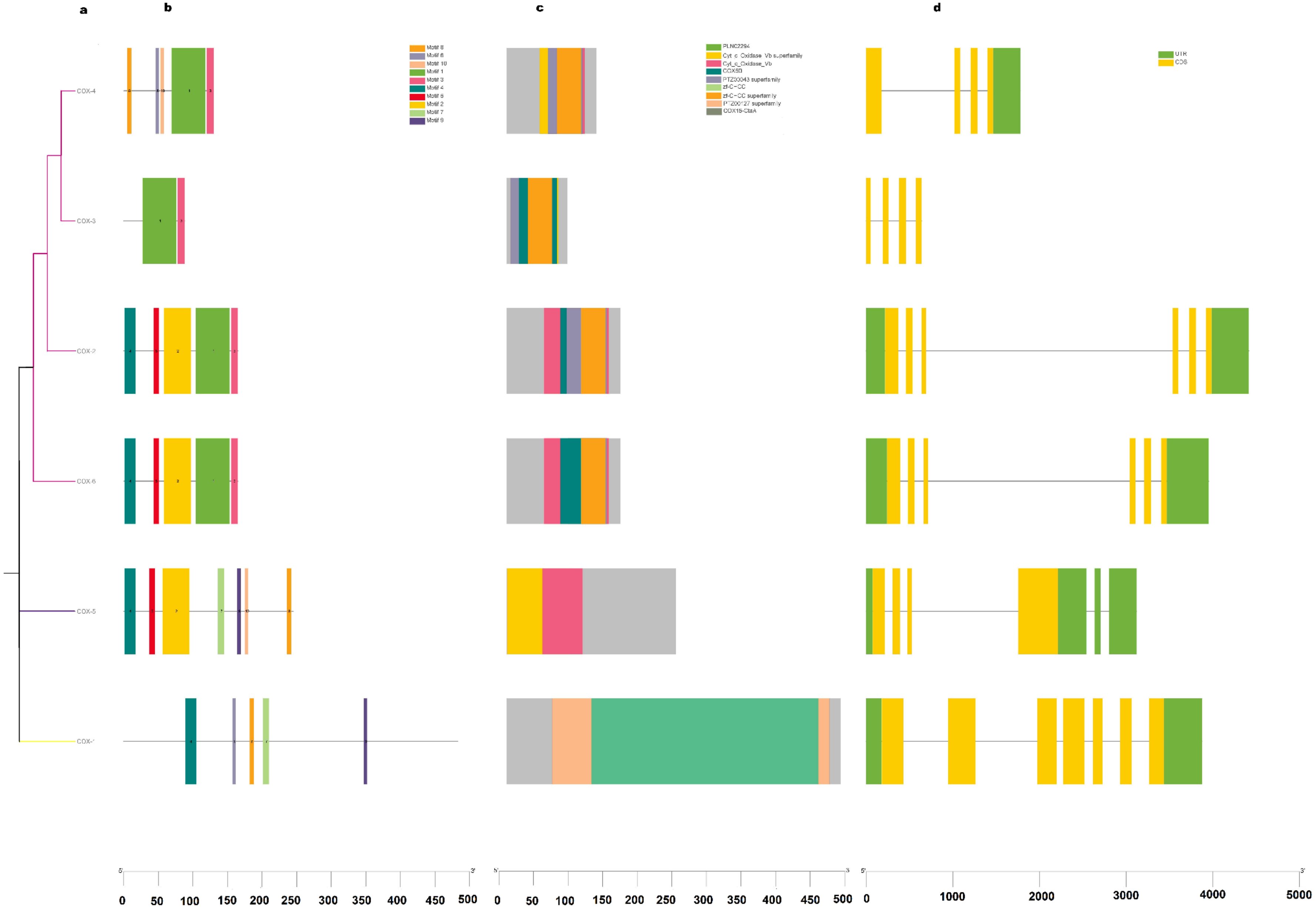

Fig. S2. COX proteins (**a**) the rectangular phylogenetic tree. (**b**) Conserved motifs were predicted using MEME. (c) Protein domains. (d) Gene structure.

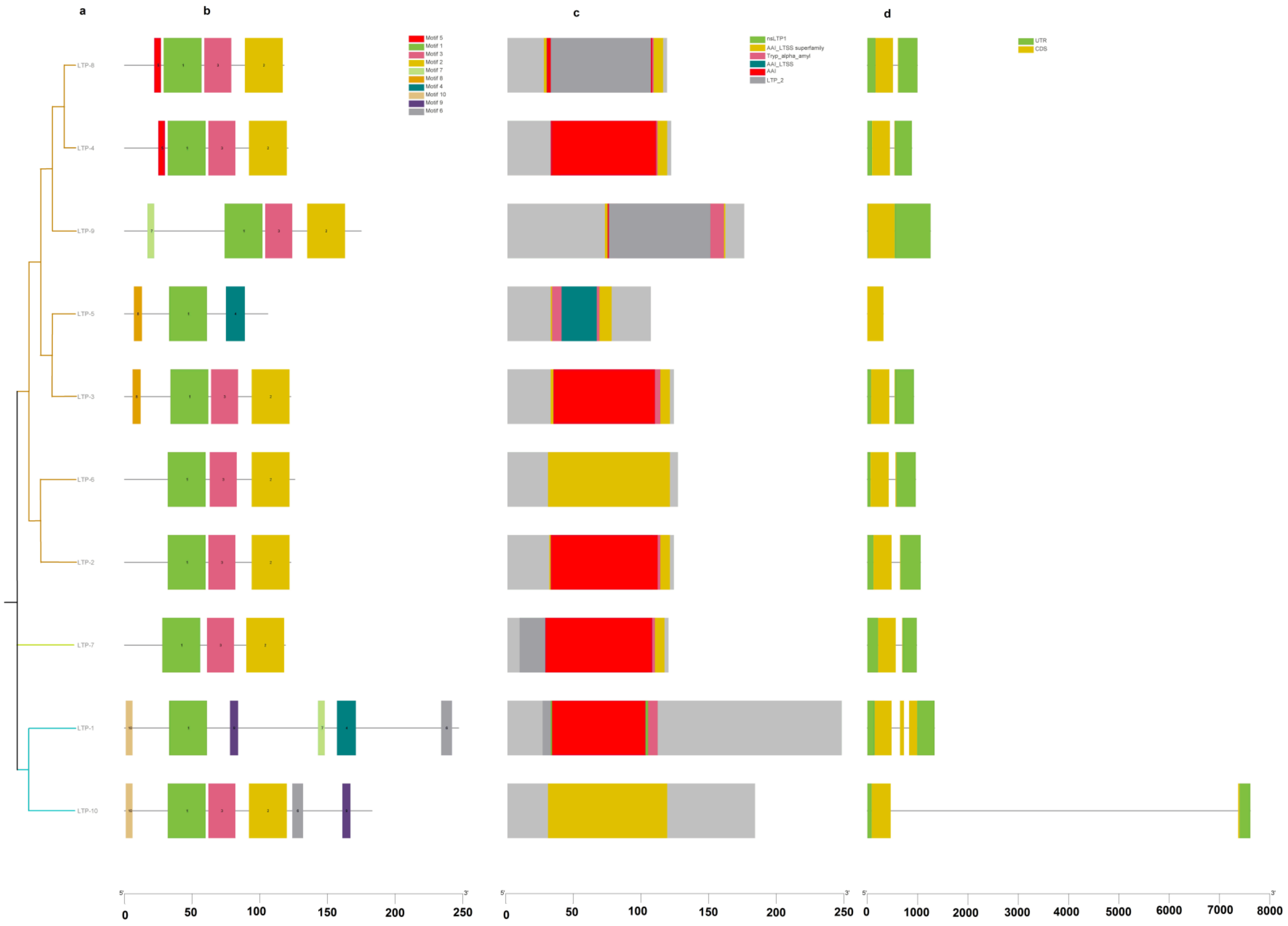

Fig. S3. LTP proteins (**a**) the rectangular phylogenetic tree. (**b**) Conserved motifs were predicted using MEME. (c) Protein domains. (d) Gene structure.

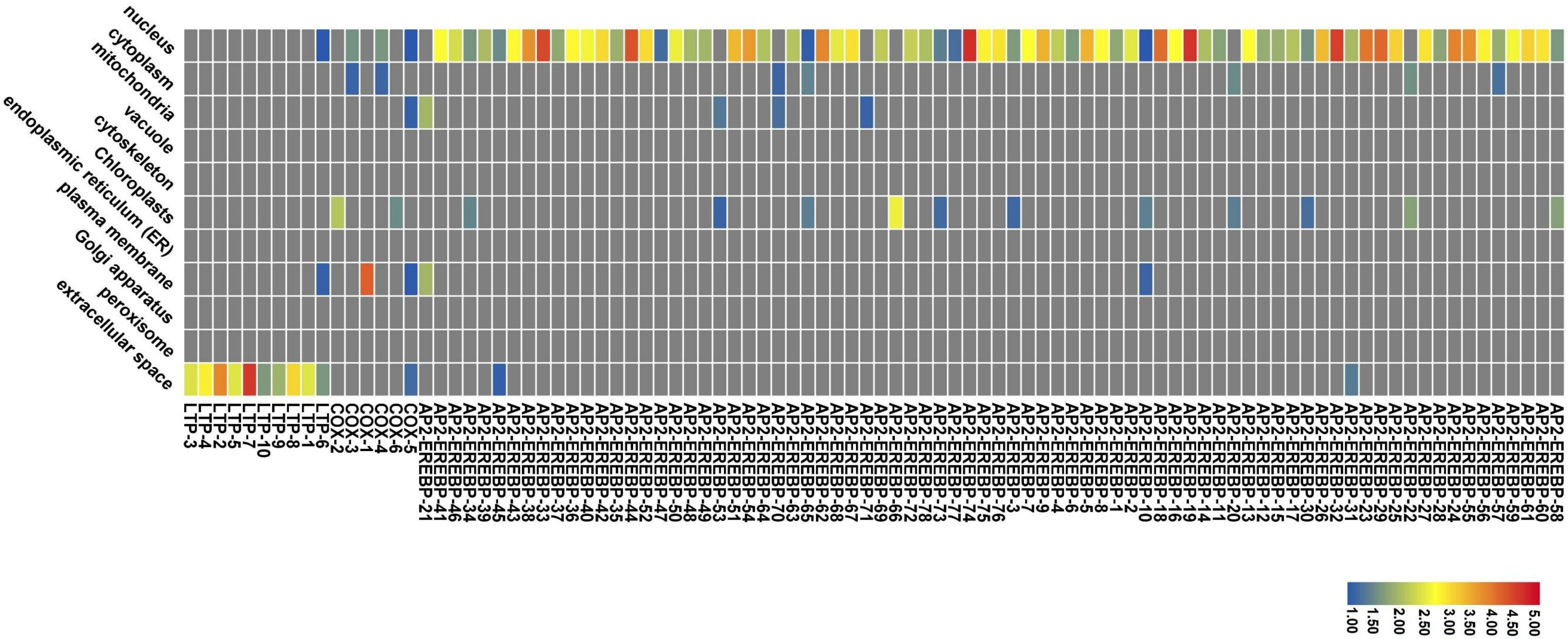

Fig. S4. Heatmap showing *AP2/EREBP*, *COX* and *LTP* proteins localization prediction in deferent organelle.

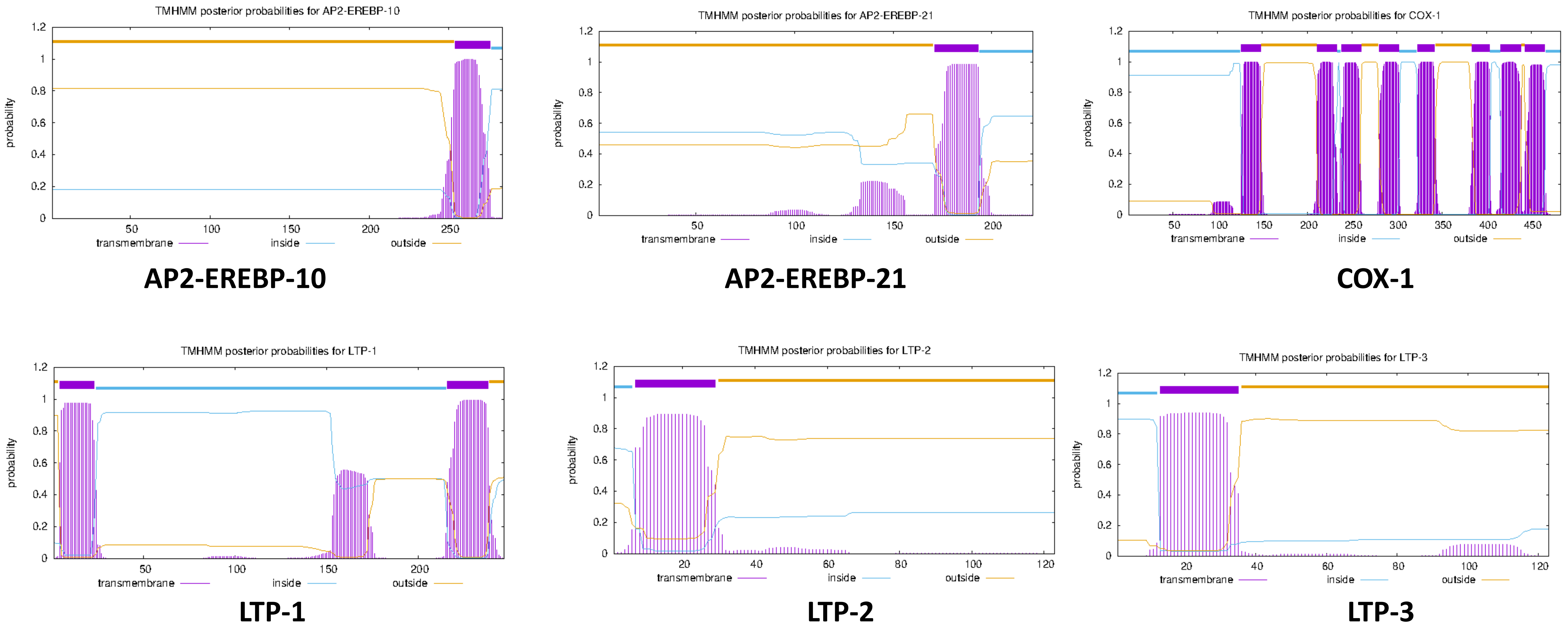

Fig. S5. The TMHMM results predicted the transmembrane helices

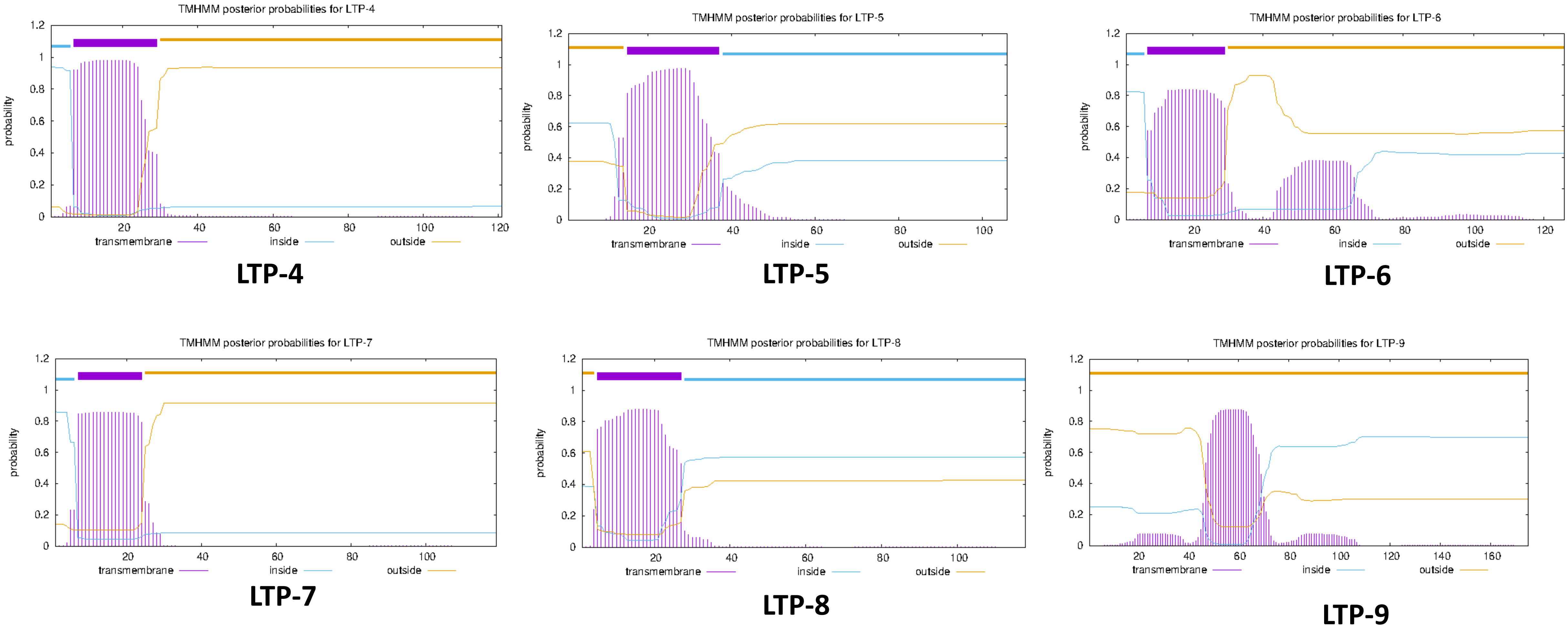

Fig. S6. The TMHMM results predicted the transmembrane helices

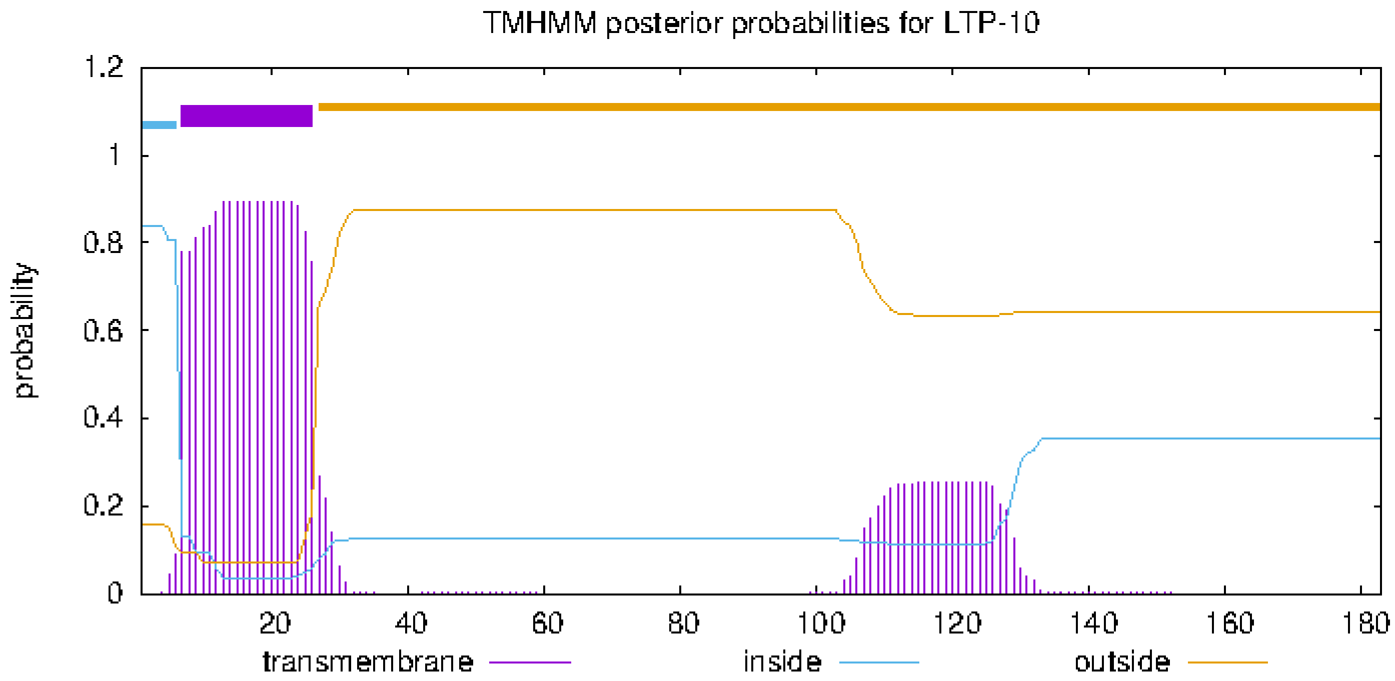

LTP-10

Fig. S7. The TMHMM results predicted the transmembrane helices

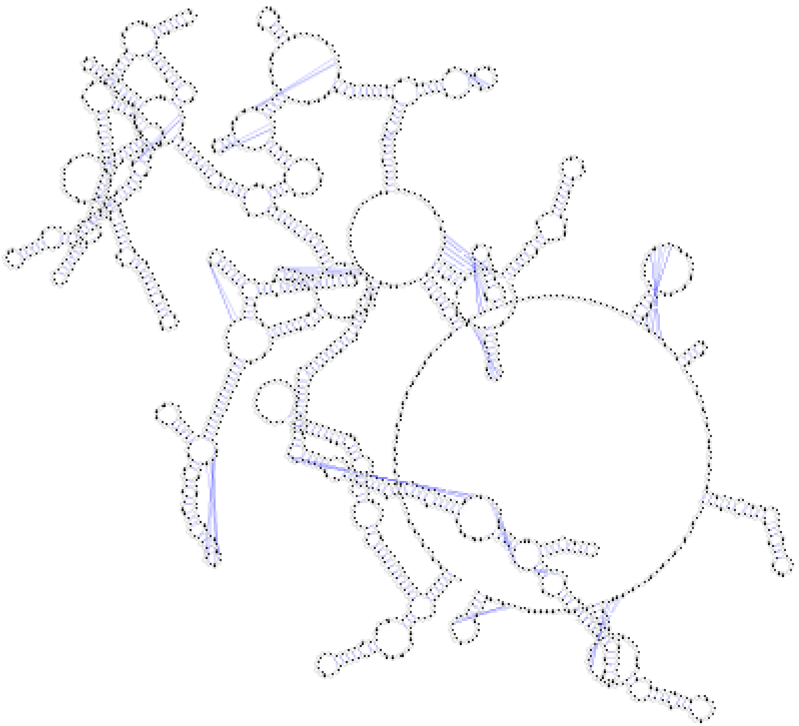

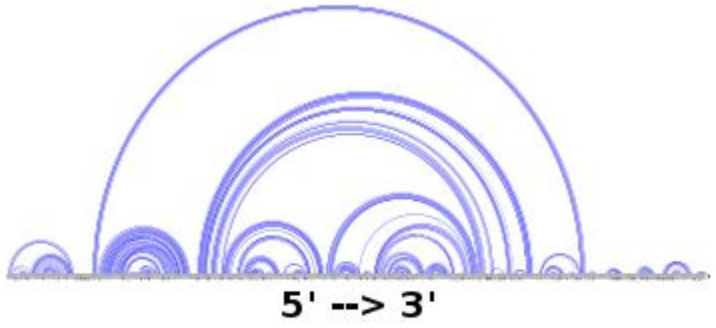

Fig. S8. Predicting RNA secondary structures of AP2-EREBP-24

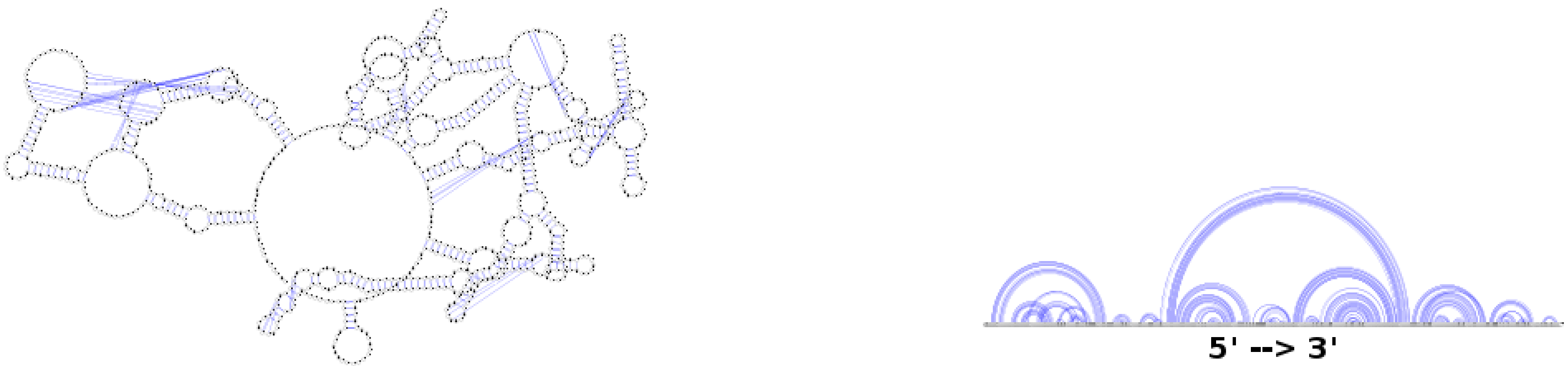

Fig. S9. Predicting RNA secondary structures of AP2-EREBP-51

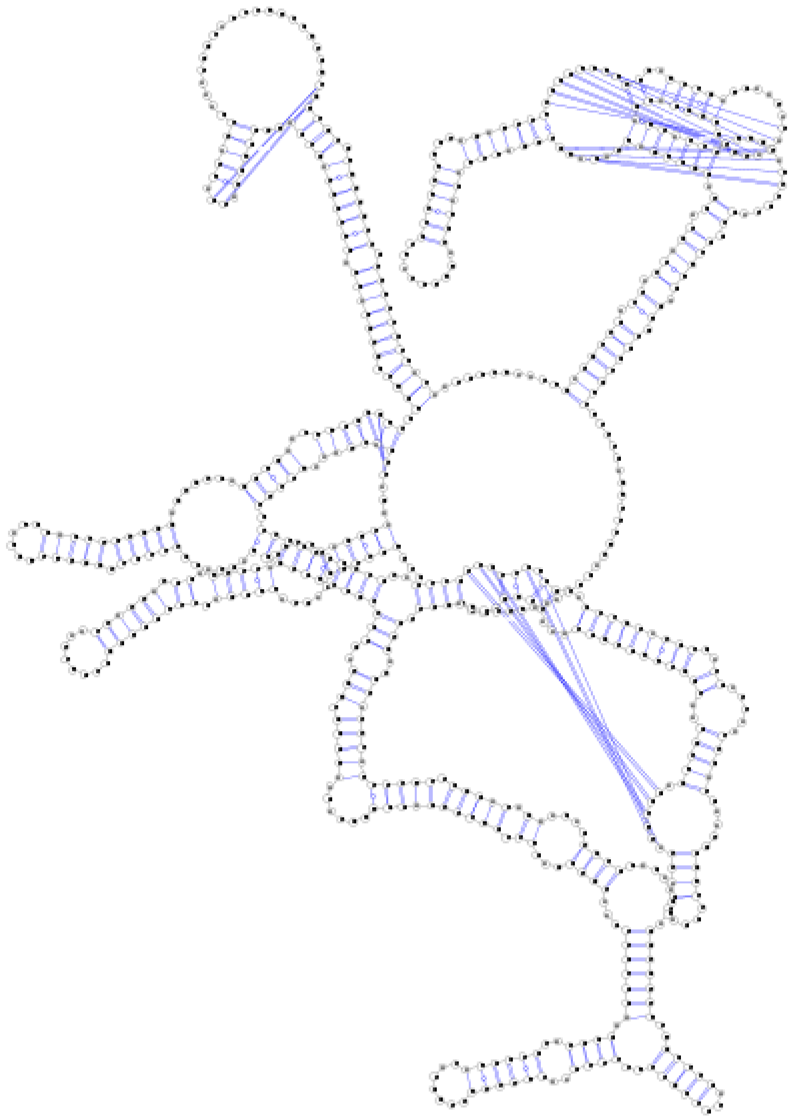

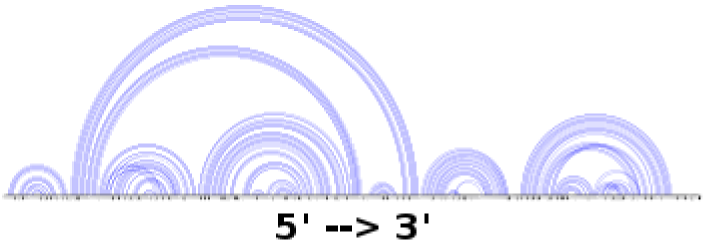

Fig. S10. Predicting RNA secondary structures of AP2-EREBP-53

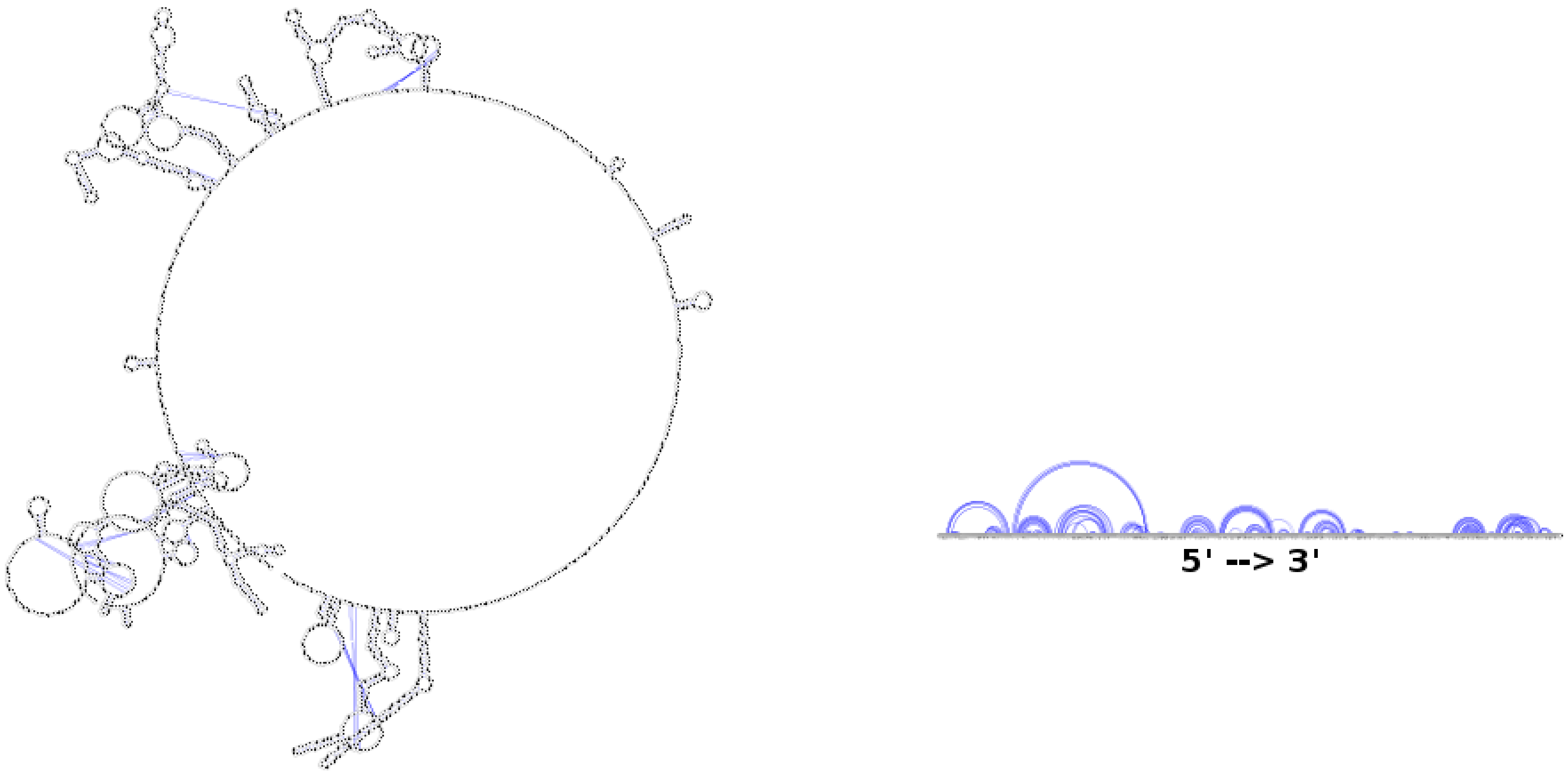

Fig. S11. Predicting RNA secondary structures of COX-1

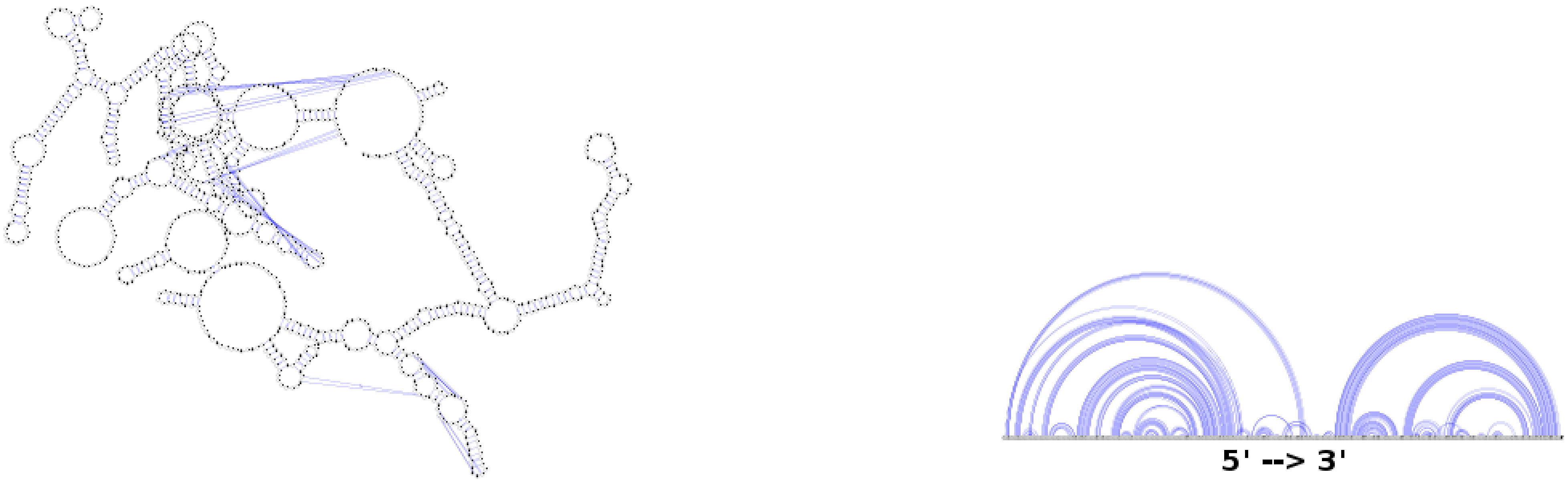

Fig. S12. Predicting RNA secondary structures of COX-2

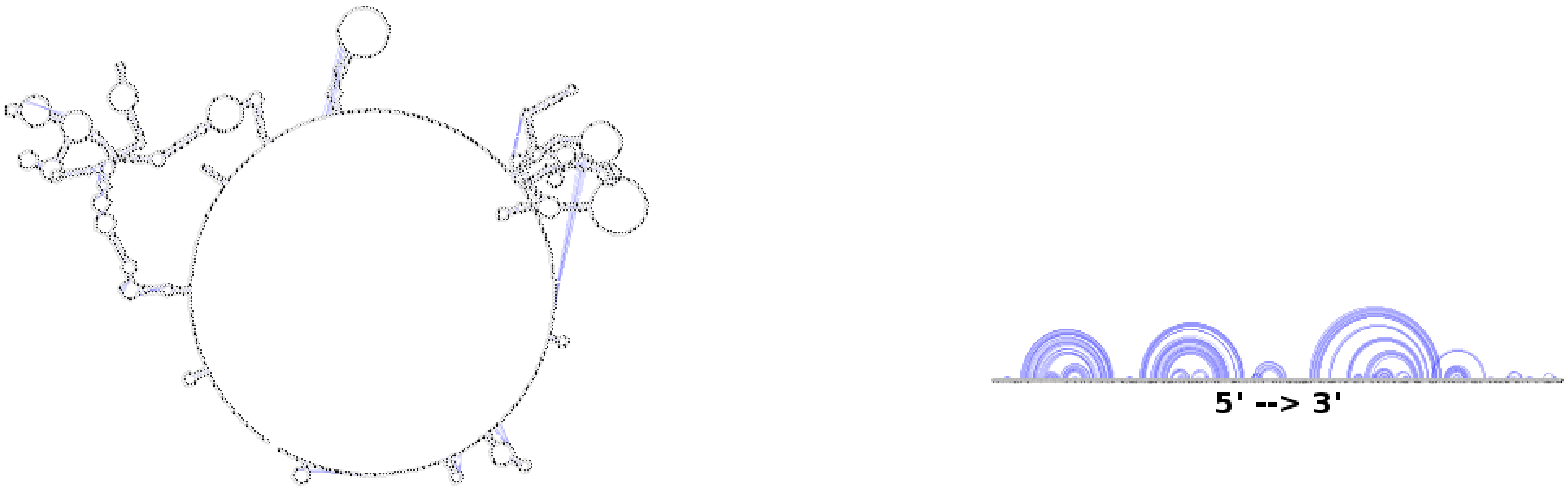

Fig. S13. Predicting RNA secondary structures of COX-5

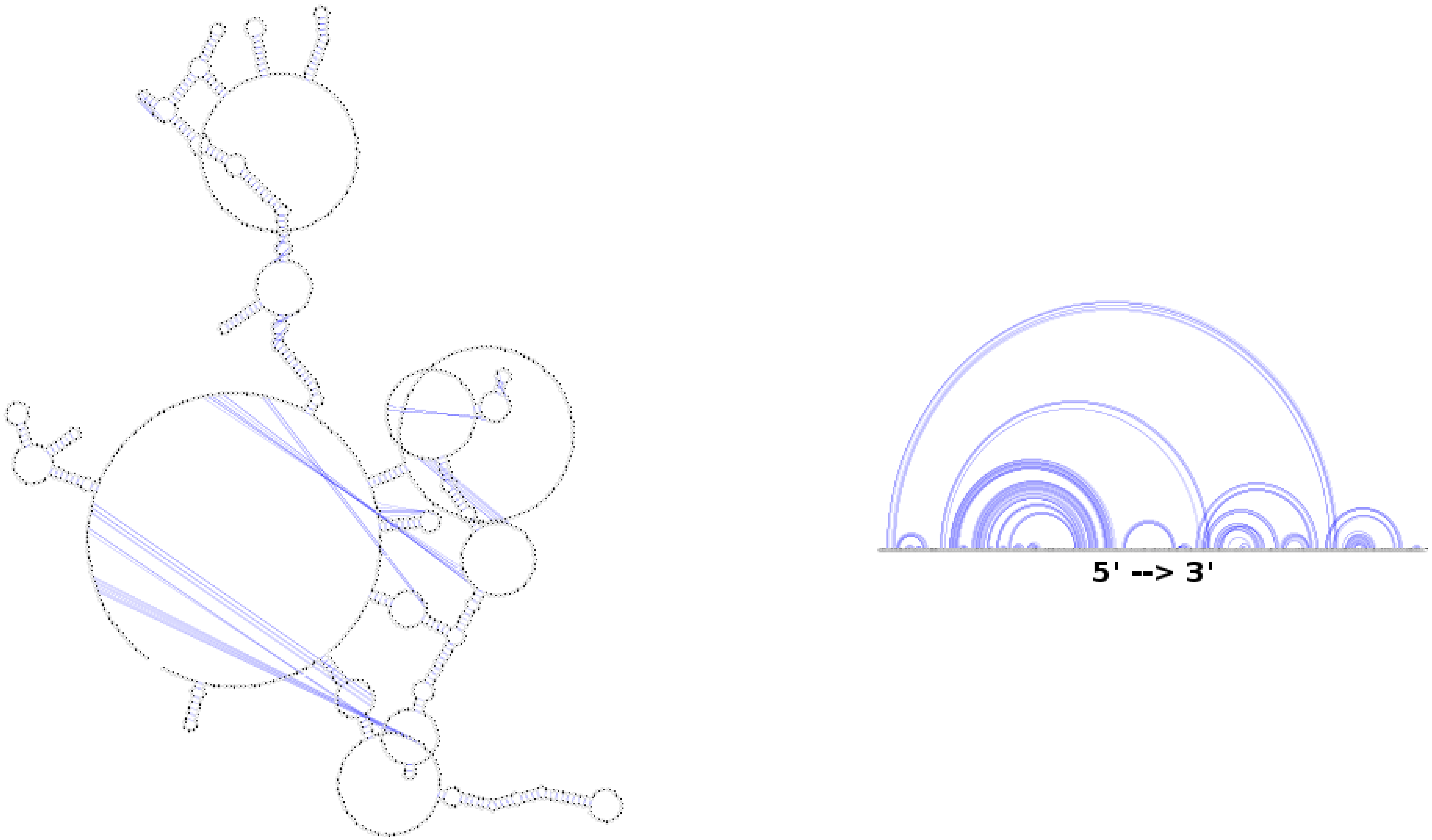

Fig. S14. Predicting RNA secondary structures of LTP-1

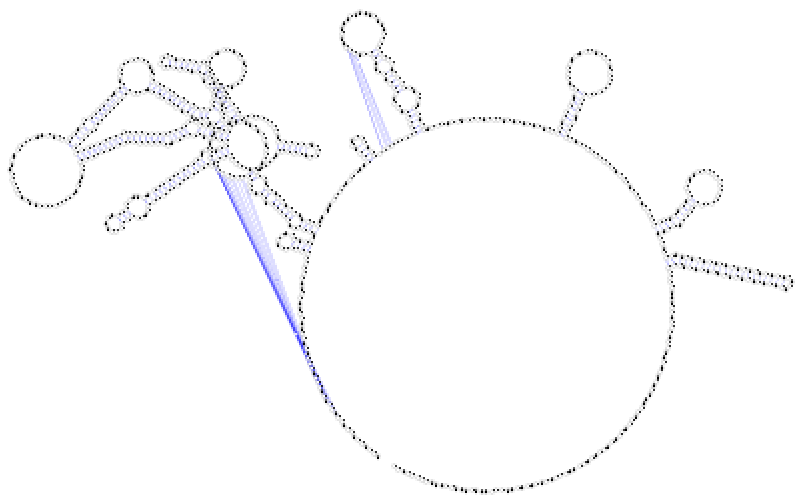

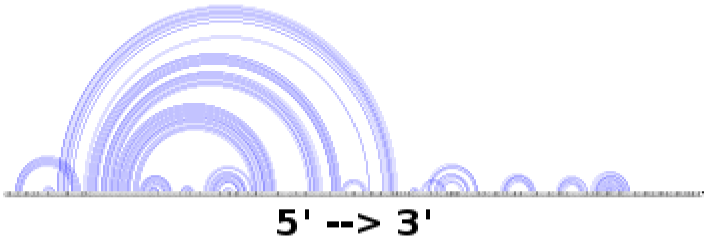

Fig. S15. Predicting RNA secondary structures of LTP-3

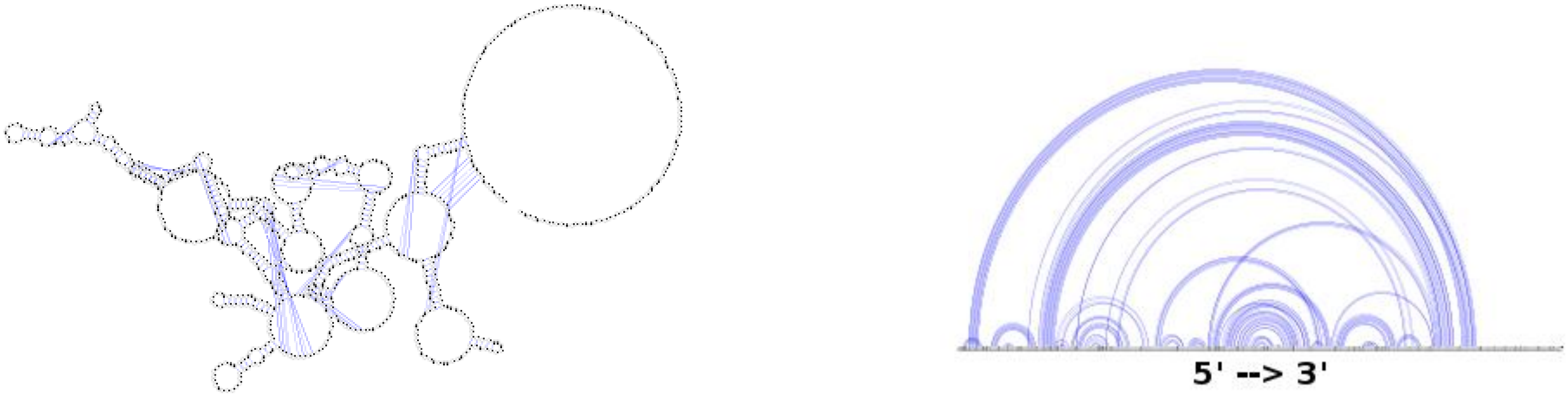

Fig. S16. Predicting RNA secondary structures of LTP-7

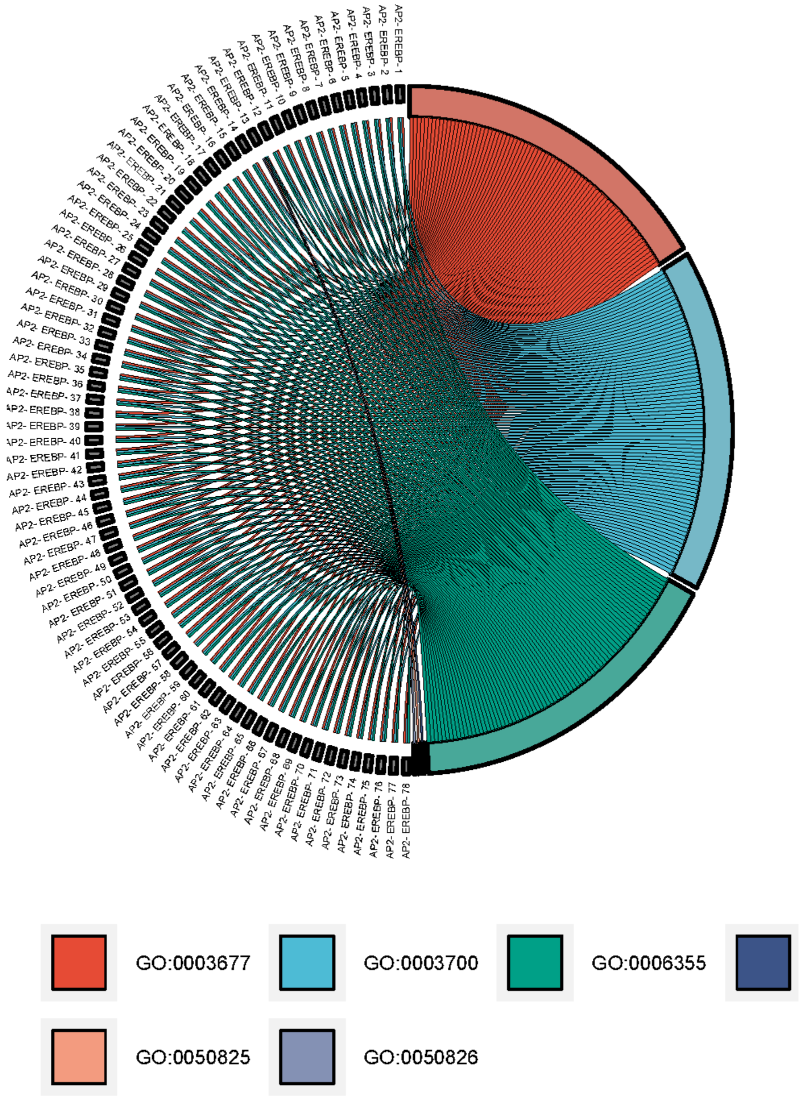

Fig. S17. GO terms for AB2-EREBP

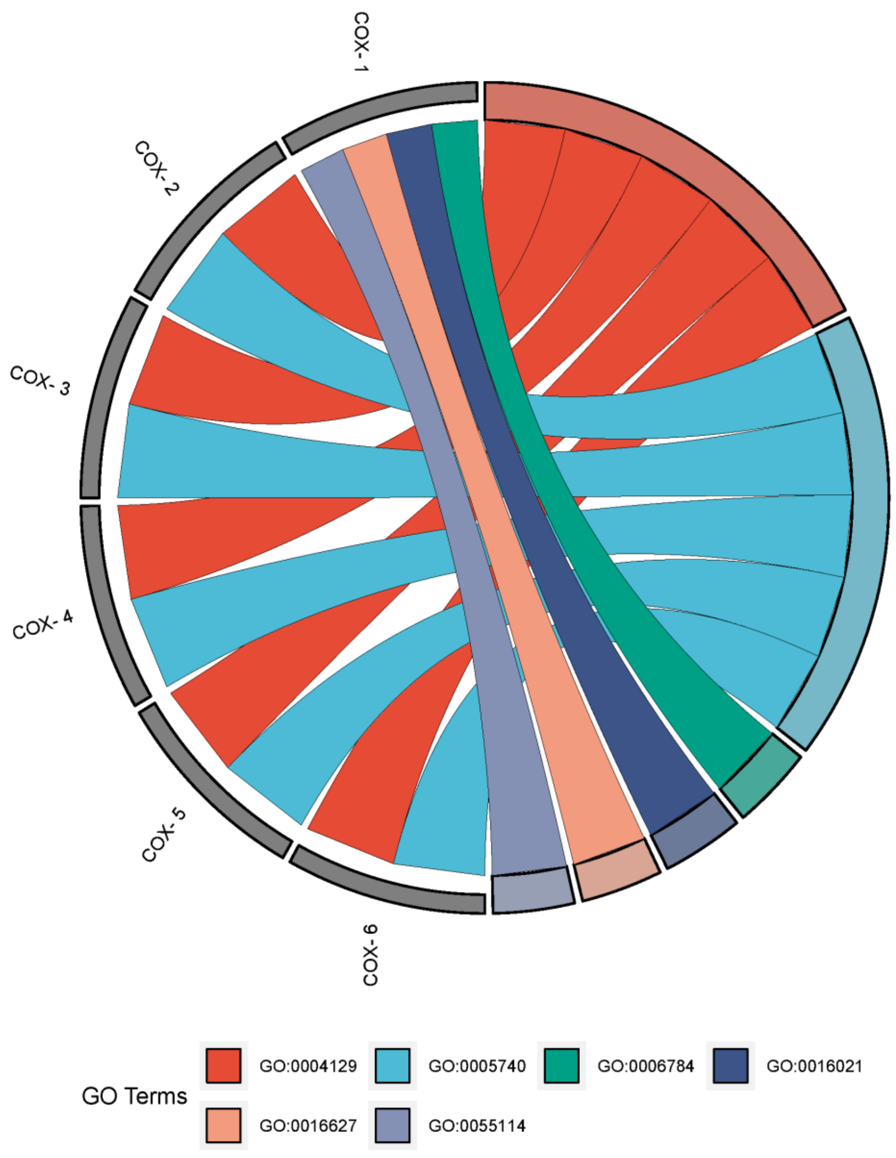

Fig. S18. GO terms for COX

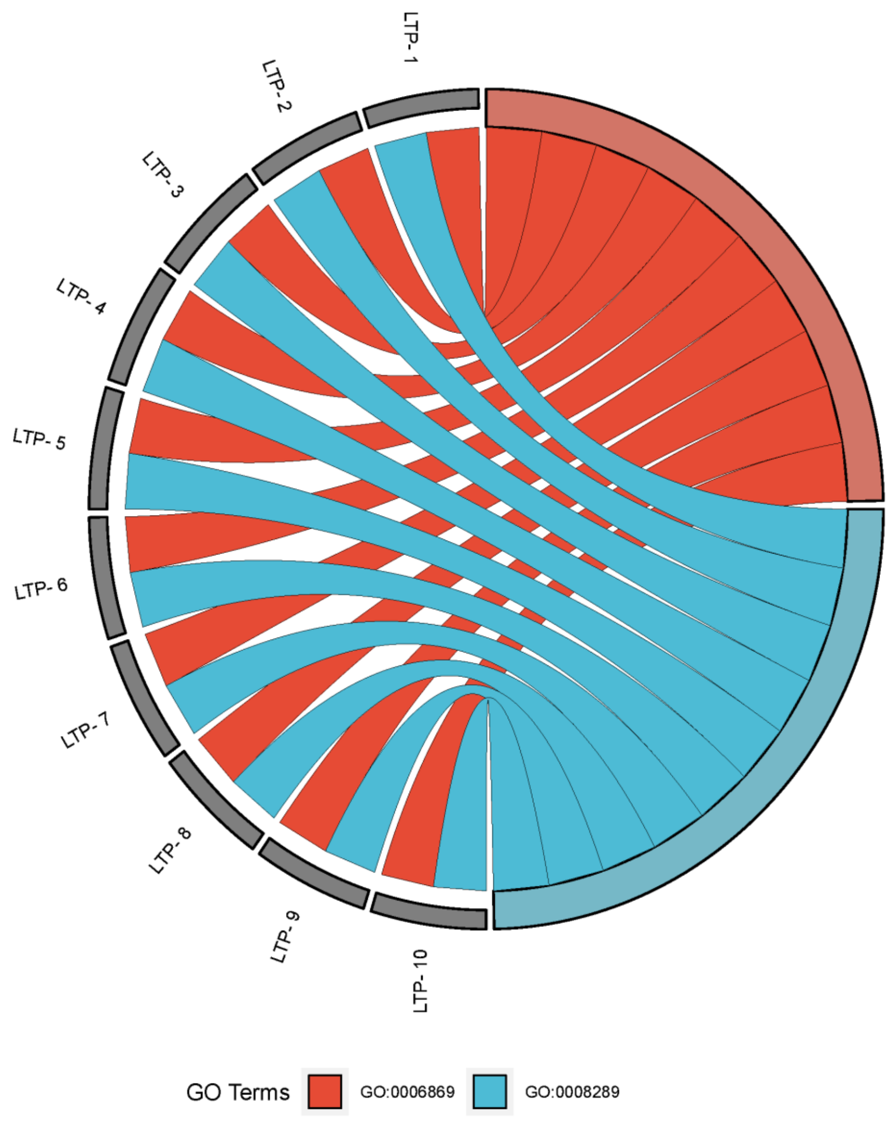

Fig. S19. GO terms for LTP

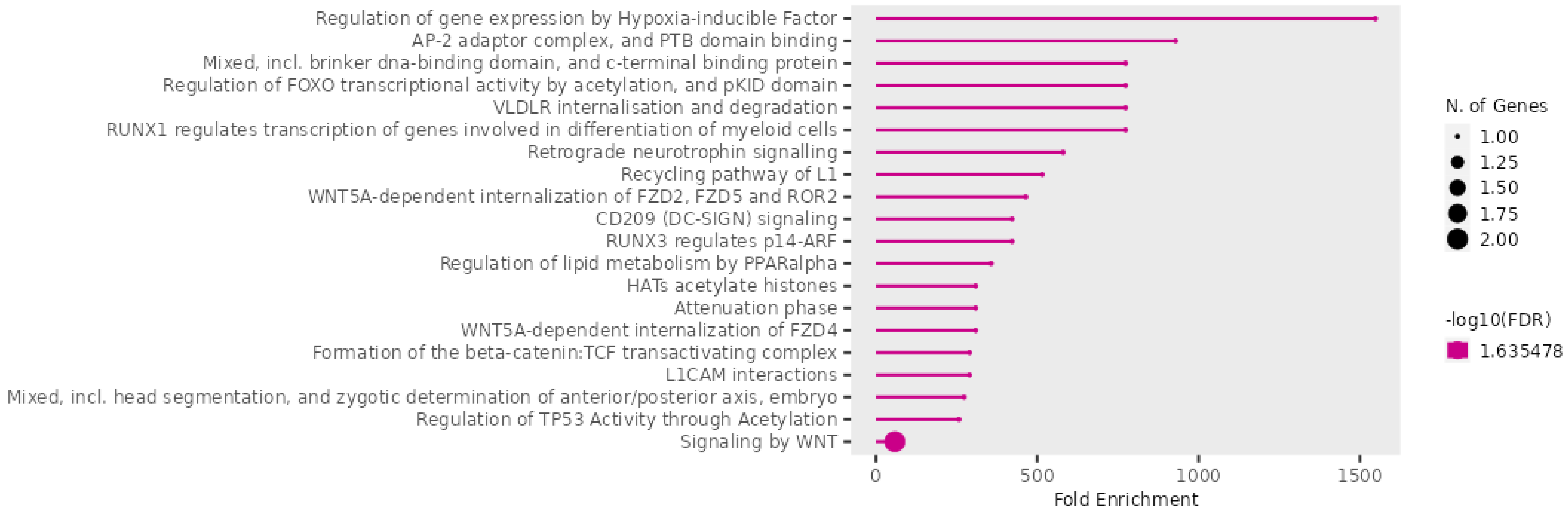

Fig. S20. Gene ontology enrichment analysis were confirmed the functional role of AB2/EREBP as stress-responsive

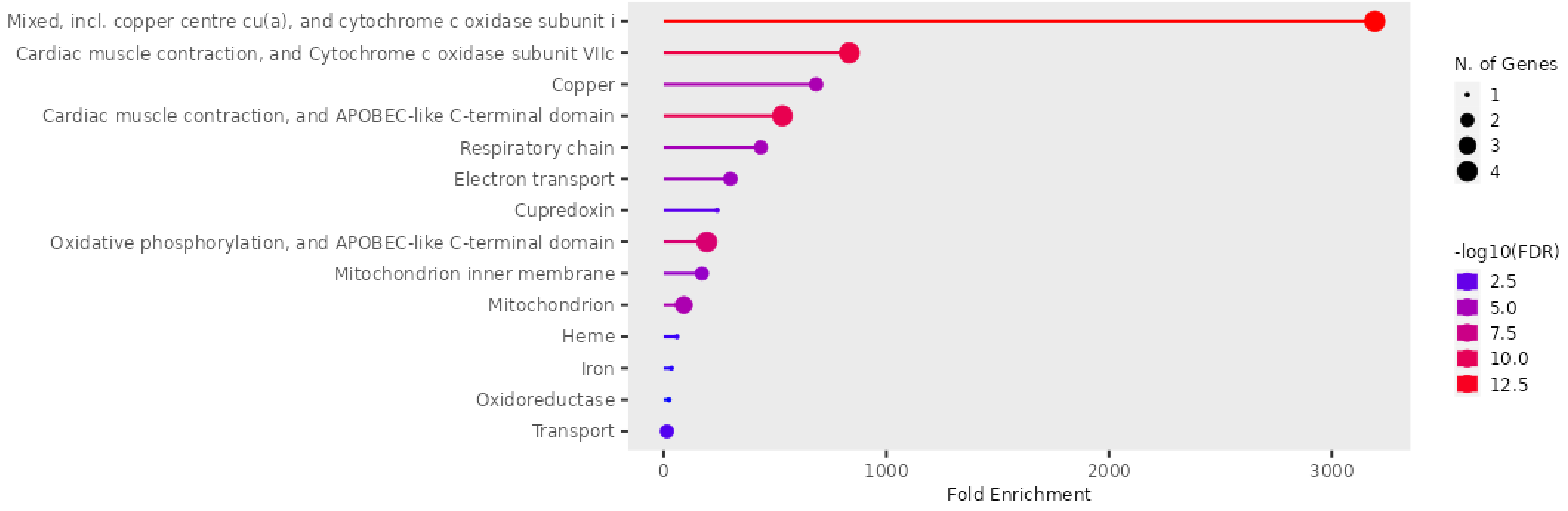

Fig. S21. Gene ontology enrichment analysis were confirmed the functional role of *COX* as a stress-responsive

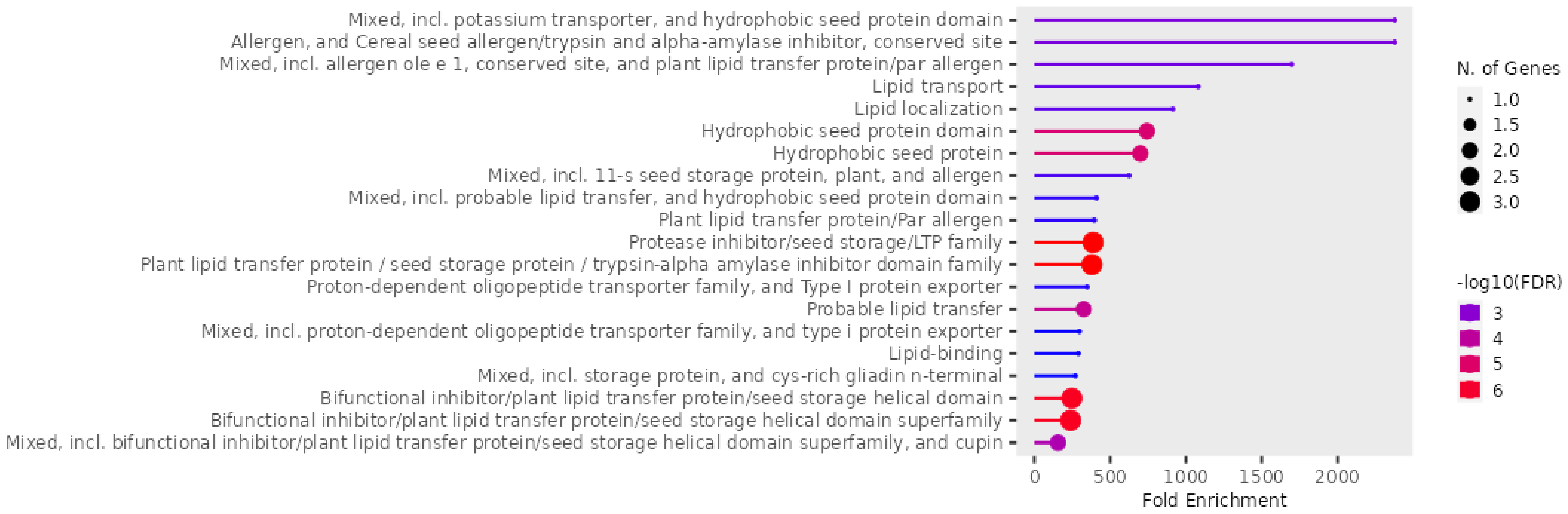

Fig. S22. Gene ontology enrichment analysis were confirmed the functional role of LTP as a stress-responsive
